# Referrals between Public Sector Health Institutions for Women with Obstetric High Risk, Complications, or Emergencies in India – A Systematic Review

**DOI:** 10.1371/journal.pone.0159793

**Published:** 2016-08-03

**Authors:** Samiksha Singh, Pat Doyle, Oona M. Campbell, Manu Mathew, G. V. S. Murthy

**Affiliations:** 1 Indian Institute of Public Health-Hyderabad, Public Health Foundation of India, Hyderabad, Telangana, India; 2 Department of Non-communicable disease Epidemiology, Faculty of EPH, London School of Hygiene and Tropical Medicine, London, United Kingdom; 3 Department of Infectious disease Epidemiology, Faculty of EPH, London School of Hygiene and Tropical Medicine, London, United Kingdom; 4 Medical Independent Consultant, Uttarakhand, India; Taipei Medical University, TAIWAN

## Abstract

Emergency obstetric care (EmOC) within primary health care systems requires a linked referral system to be effective in reducing maternal death. This systematic review aimed to summarize evidence on the proportion of referrals between institutions during pregnancy and delivery, and the factors affecting referrals, in India. We searched 6 electronic databases, reviewed four regional databases and repositories, and relevant program reports from India published between 1994 and 2013. All types of study or reports (except editorials, comments and letters) which reported on institution-referrals (out-referral or in-referral) for obstetric care were included. Results were synthesized on the proportion and the reasons for referral, and factors affecting referrals. Of the 11,346 articles identified by the search, we included 232 articles in the full text review and extracted data from 16 studies that met our inclusion criteria Of the 16, one was RCT, seven intervention cohort (without controls), six cross-sectional, and three qualitative studies. Bias and quality of studies were reported. Between 25% and 52% of all pregnancies were referred from Sub-centres for antenatal high-risk, 14% to 36% from nurse run delivery or basic EmOC centres for complications or emergencies, and 2 to 7% were referred from doctor run basic EmOC centres for specialist care at comprehensive EmOC centres. Problems identified with referrals from peripheral health centres included low skills and confidence of staff, reluctance to induce labour, confusion over the clinical criteria for referral, non-uniform standards of care at referral institutions, a tendency to by-pass middle level institutions, a lack of referral communication and supervision, and poor compliance. The high proportion of referrals from peripheral health centers reflects the lack of appropriate clinical guidelines, processes, and skills for obstetric care and referral in India. This, combined with inadequate referral communication and low compliance, is likely to contribute to gaps and delays in the provision of emergency obstetric care.

## Background

Worldwide it is estimated that 287,000 women die due to maternal causes every year [1]. The majority of maternal deaths are due to direct obstetric causes [2]. Most obstetric complications, except those abortion-related, occur during delivery or immediately after delivery, and they have the potential to rapidly become life threatening [3]. To prevent maternal deaths, the complications occurring at home or birthing centres require timely and appropriate referral to basic emergency obstetric care (BEmOC) or specialist comprehensive emergency obstetric care (CEmOC), and referral from BEmOC to CEmOC [4,5,6,7]. A systematic review on interventions for improving maternal health observed that most successful programs focused on training for CEmOC, the placement and motivation of care providers, refurbishment of existing health institutions and establishment of referral and transportation systems [8].

An efficient referral system provides access to treatment and skills by linking different levels of care through appropriate referrals [9]. To refer a patient is a medical decision and depends on many things including the skills of the referring staff, the tools for diagnosis, the availability of a health institution with specialist facilities, the quality of care at the referral institution, the cost of care, distance, transportation, communication, someone to travel with the patient, and feasibility of travel by the patient [7]. The type of obstetric complication determines the level of care needed and the place to be referred to, and this makes the referral pathways complex [7,10]. Compliance to referral may depend on the counselling skills of the referrer, the socio-cultural beliefs of the patient and her family, and perceptions of the quality of care [7]. A recent systematic review on interventions to improve referral systems and transportation for EmOC in developed country settings observed that most programs focus on birth preparedness, complication readiness, availability of transport, and costs of transport [10]. The interventions included in the review focused on improving self-referrals, with only a few on interventions to improve referrals between institutions, the number and quality of institution-referrals, and transport for between-institution transfers.

India accounts for a fifth of annual global maternal deaths (56,000) [1] and the Maternal Mortality Ratio (MMR) is estimated to be 167/100,000 live births (Sample Registration System, SRS-2011-13) [11]. India has implemented many interventions to reduce the MMR, including schemes to strengthen health infrastructure and to improve the proportion of institutional deliveries [12]. There is some evidence that these schemes increased institutional deliveries [13,14] but a corresponding reduction in the MMR was not achieved [15–17]. Maternal death reviews from India suggest that most of the mothers who died had gone through multiple referrals before reaching the appropriate facility [18–20].

A preliminary review of Indian health policy, Reproductive Health Program documents and interview with state maternal health consultants, by reviewers, revealed that there are no standard procedures or referral protocols for obstetric emergencies and complications in India. SBA training manuals mention clinical criteria for referral but these guidelines are not supported by appropriate resources in the health system. Usually no records relating to referrals between institutions are kept and no referral slips or communication about the referred case is provided to the next level institution [21]. There is no routine feedback mechanism or routine monitoring of the appropriateness of referrals in India [21].

### Rationale for Systematic review

There is a paucity of evidence from India on the proportion of complicated and emergency obstetric cases that are detected at the primary health institution level and referred to appropriate higher level health institutions. This systematic review from India will help understand the existing referral criteria, referral pathways, factors affecting referrals and proportion of referrals for obstetric care across the country. With changing policies and interventions to strengthen EmOC it is necessary to understand the changes in referral systems over time and existing needs in India.

### Research Question

What is the proportion of referrals between public health institutions for women with obstetric high risk, complications, or emergencies in India?

#### Secondary question

What are the socio-economic and medical characteristics of women who are referred for obstetric causes and what are the referral pathways utilized?

## Methods

The research obtained ethics approval from ethics committees of both LSHTM and IIPH-Hyderabad. (LSHTM Ethics Ref: 9613; IIPHH Ethics Ref: IIPHH/TRC/IEC/009/2014)

### Summary of the health care system in India

**Fig 1:**
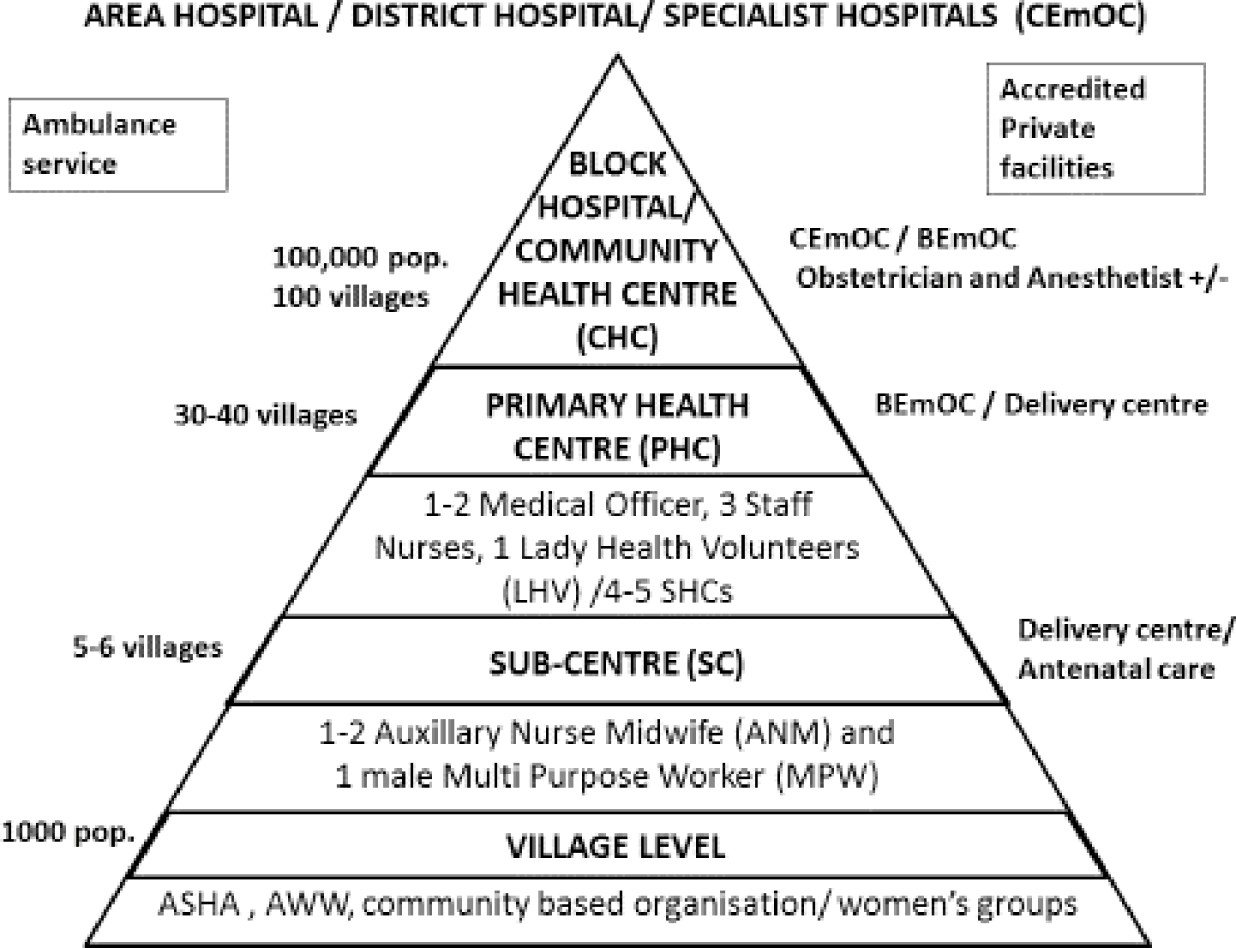
Rural public health system for obstetric care in India.

The Subcentre (SC) is the most peripheral unit in the existing government health care system in rural India and is the first level of contact where antenatal care is provided (Fig 1). In a few states deliveries are also conducted at SCs by a trained Auxillary nurse midwife (ANM)/ Health worker (Female) or nurse. Primary health centres (PHCs), the next level, have been proposed as 24X7 BEmOCs but many just work as delivery centres and a few do not even provide delivery services. Community health centres (CHCs) are where an obstetrician may be present and a CHC may work as a BEmOC or a CEmOC. First referral units (FRUs) are upgraded CHCs, Sub-district hospitals, District hospitals and specialist hospitals able to provide CEmOC care [22]. Pregnant women can choose to go directly to any of these centres, by-passing the hierarchy. In case of referral they can choose to comply with referral advice or go elsewhere.

In urban areas, there are dispensaries, urban health centres and urban health posts which provide antenatal care and referral. A few of the urban health centres have been upgraded as delivery centres. At the next level are Maternity hospitals (BEmOC) and secondary and tertiary care hospitals (BEmOC/CEmOC) [23]. There are several medical colleges and other hospitals that provide specialised care. These are mostly located in urban areas and cater to the referral needs of both rural and urban populations.

### Working Definitions

An institution-referral is when a client seeks care at a lower level health institution (delivery centre or BEmOC) and is referred onwards to a higher level institution (BEmOC or CEmOC) by health staff for specialist attention. The referral is made for reasons of a high risk pregnancy, complications during pregnancy and puerperium, or an emergency at any time in pregnancy and puerperium.

### Search Strategy

The literature search was conducted using six mainstream databases (Medline, Embase, Popline, IMSEAR, Cochrane Central Register of Controlled Trials (CENTRAL) and CINAHL) and four other databases (WHO, UNICEF, UNFPA and Indian RCH repository). Grey literature sources, such as program reports, were also used. The review was restricted to studies from India, published in English between 1994 and 2013.

The electronic search strategy was based on terms related to referral or emergency, and pregnancy, obstetric high risk, obstetric complications, or obstetric emergencies, and India. Appropriate MeSH and/or keywords using respective thesauri were used in the search strategy as mentioned in S1 Text.

#### Inclusion criteria

1. All studies (hospital or community based) and reports with institution-referrals for obstetric care, with any kind of epidemiological study design, were included. Studies reporting either in-referrals from lower-level institution or out-referrals to higher-level institution were included.
2. Type of participants in the studies or reports: Studies with pregnant, post-abortion and post-partum women referred by staff from designated public health institutions to a higher level referral institution were included.
3. Place of study: India

#### Exclusion criteria

1. Studies or reports on referrals for non-maternal conditions.
2. Editorials, commentaries and letters.

### Screening

Screening was done by two independent researchers using the inclusion and exclusion criteria. Screening was first done based on titles and abstracts and then subsequently by reading full text. Disagreement between reviewers, was resolved by discussion and establishing consensus.

### Data Collection

Measurement indicators studied were: the proportion of in-referrals and out-referrals; cause-specific referrals; place from where referred and the place referred to; pre-hospital treatment; availability and arrangements for transportation; type of transport and communication; costs and cash incentives; and compliance rates, and socio-economic and medical characteristics of women referred. Data extraction forms were developed and piloted before use. Information was extracted on the type of intervention, if any, and the prevalence of outcomes and costs were considered. Key qualitative findings were also recorded and described.

The quality of papers was assessed using STROBE guidelines for observational studies and CONSORT guidelines for intervention studies. A score of 1 was assigned to each item in the checklist and a total score was calculated for each paper (maximum score- STROBE = 22; CONSORT = 25). A score below 11 out of 22 for observational studies, and below 13 out of 25 for intervention studies, indicated poor quality. Potential risk of bias in methods (selection, performance and detection), analysis and reporting were assessed for each of the studies with respect to study designs. Reviewers also discussed the limitations in combining the results from different studies in the review.

### Synthesis of results

Both quantitative and qualitative research studies were included. Each selected study was assessed with respect to the type of study and measurement indicators (proportions). Findings were summarized separately for a) abortion and post-abortion care, b) antenatal high-risk and c) complications and emergencies any time during pregnancy and the puerperium. Qualitative studies were reviewed to provide supplementary information regarding institution-referral rates, pathways and barriers to appropriate referral. The review was reported in line with the PRISMA checklist as reported in S1 PRISMA Checklist.

## Results

### Search results

**Fig 2:**
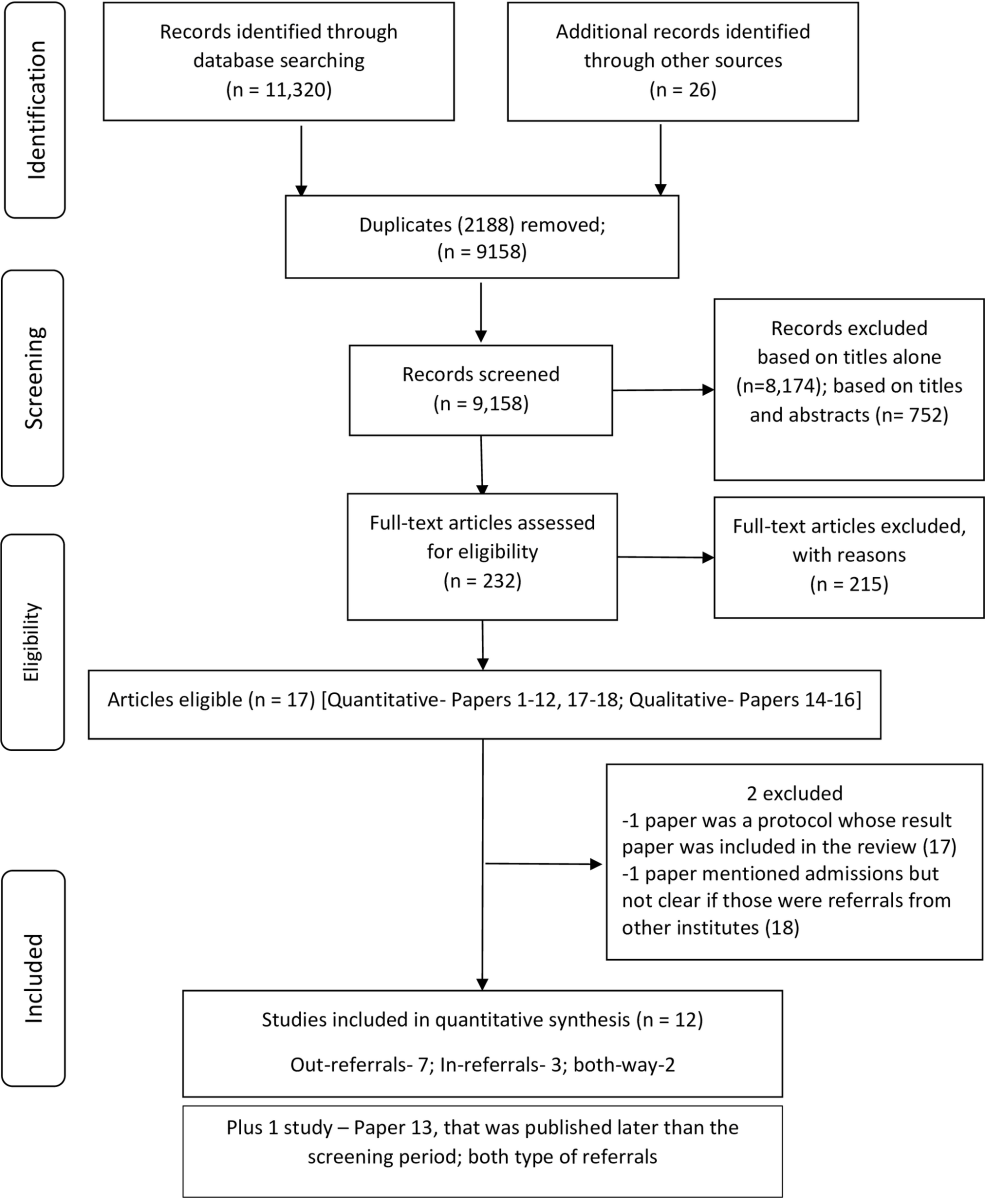
PRISMA Flow Diagram for Systematic Review.

The search yielded 11,346 articles from electronic searches and other sources (program or project reports from specified organisational repositories) (Fig 2). Duplicates (2,188) were removed before screening. A total of 9,158 articles and reports were screened for eligibility, of which 8,174 were excluded based on titles alone and 752 based on titles and abstracts. Reasons for exclusion were mainly that the studies were not from India, were not about pregnant women or pregnancy, or were editorials. A total of 232 articles and reports were selected and full texts were read to assess for inclusion. Of these, 215 articles were excluded because they did not mention institution-referral pathways or proportions of institution-referrals. Finally, three qualitative articles and fifteen quantitative research articles were found eligible for inclusion [24–41]. Of these, two articles were from the same study: one was a study protocol [40] whose subsequent article on results [32] was included for quantitative analysis. One article was further excluded at the time of data extraction [41]. Although this article mentioned admissions in hospital as referrals, it could not be concluded if those were referrals from other institutions. During the synthesis of results and reviewing new literature, researchers found one paper very relevant to the review but it was published in 2014 i.e. later than the search criteria of up to 2013 [36]. It was decided to include this paper in the results.

### Characteristics of included studies

#### Quantitative

Out of the thirteen studies, out-referrals from a health institution was documented in 7 (papers 1–3, 6–9, Table 1), in-referrals to a health institution in 3 (papers 10–12, Table 1) and both out-referrals from and in-referrals to a health institution were documented in 3 studies (papers 4, 5, 13, Table 1). Among the 7 out-referral studies, five were prospective cohort studies following an intervention (without controls), one was a cluster randomised trial, and one a cross-sectional study. Two of the three studies that mentioned both-way referrals were prospective cohort studies following interventions and one was a cross-sectional study. All 3 in-referral studies were cross-sectional studies. One of these cross-sectional studies was only about abortions [35]. Characteristics of the studies are described in Table 1.

**Table 1:** Characteristics of studies included in the review.

SNo	Author	Type of study	Time of study	State	Rural/ Urban	Type of institutions	Participants (N0.s)	Intervention
**Out-referrals**								
1	Maitra 1995 [24]	Intervention- Prospective Cohort^b^	1987–1990	Uttar Pradesh, Madhya Pradesh, Haryana, Rajasthan, Gujarat, Maharashtra	Rural	SC and PHC	Antenatal women registered at SC or PHC (12,907)	Training of community for high-risk; Training of ANMs and MO for ANC, high-risk screening, referral and record keeping for referrals
2	Hitesh 1996 [25]	Intervention–Prospective Cohort^b^	1993	Rajasthan	Rural	SC	Antenatal women in community (206)	Training of ANMs and TBAs for ANC, high risk screening and referral. Red referral card was issued to refer women.
3	McCord 2001 [26]	Intervention–Prospective Cohort^b^	1996–1999	Maharashtra	Rural	Community and Private hospital	Antenatal women and women in labour in the community (2,905 pregnancies)	Training of community via VHWs; Training of VHWs for ANC, high risk screening, delivery care, complication identification and referral; low cost delivery and referral care at private hospital
4	Barua 2003^a^ [27]	Intervention–Prospective Cohort^b^	1994–2001	Maharashtra	Rural	Community and PHC	Antenatal and postnatal women attending clinics (NA)	Training of ANMs for ANC, high risk screening and referral to PHC, establishing ANC clinics to be run by ANMs, MOs of PHC trained for supervision and referral to DH.
5	Iyengar 2009^a^[28]	Intervention–Prospective Cohort^b^	2000–2008	Rajasthan	Rural	Equivalent to PHC run by NGO midwife / nurse	Antenatal, intra-natal and postnatal women attending at the health institution (2,771 deliveries + 400 in-referred complications)	Training of nurse midwifes at health institution for ANC, EmOC and referral in consultation with on-call obstetrician
6	More 2010 [29]	Cross-sectional	2005–2007	Maharashtra	Urban slums	Community	Pregnant women who delivered in the community (10,754)	-
7	David 2012 [30]	Intervention- Retrospective Cohort^b^	2005–2010	Tamil Nadu	Urban	UHC	Antenatal, intra-natal and post-natal women at the health institution (1,873 deliveries)	Training of 2 nurses at UHC for ANC, EmOC and referral in consultation with on-call family physician
8	Alehagen 2012 [31]	Intervention- Prospective Cohort^b^	2006–2009	Maharashtra	Rural	Community, SC and PHC	Antenatal women and women in labour in the community (31,693 deliveries)	Training of community for high risk & complication via female health volunteers; Training of ANMs and TBAs for ANC, high risk screening and referral; Training of ANMs and TBAs for safe delivery at home or PHC, complication identification and referral; Training of Nurses and MO at PHC for supervision. Establishing 9 PHCs and 5 mobile clinics.
9	Pasha 2013 [32]	Cluster RCT	2009–2011	Maharashtra, Karnataka	Rural	Community, PHCs and referral hospitals	Antenatal women and women in labour in the community (20,852 deliveries in Intervention; 18,551 in control)	Training of community via community facilitators for high risk, complication and birth preparedness; Training of community birth attendants (TBAs and ANMs) for home based life-saving skills and referral; Training of staff at health institution for EmOC facility improvement.
**In-referrals**								
10	Biswas 2004 [33]	Cross-sectional	1997–1998	West Bengal	Rural and Urban	First Referral units(FRUs)—Area and Rural hospitals	Pregnant women admitted for delivery (26,062)	-
11	Kaul 2006 [34]	Cross-sectional	2000–2003	Chandigarh	Rural and Urban	Tertiary hospital	Postnatal women who developed PPH at the hospital or admitted with PPH after delivery (178)	-
12	Banerjee 2012^c^ [35]	Cross-sectional	2006	Madhya Pradesh	Rural and Urban	Secondary and Tertiary Hospital	Women seeking care for post abortion complications (786)	-
13	Chaturvedi2014^a^ [36]	Cross-sectional	2014	Madhya Pradesh	Rural and Urban	Secondary and Tertiary Hospital	Women seeking care for intra-natal care (1182)	Government of India managed Janani Suraksha Yogana which provides cash incentives to women delivering in institutions. Part of this incentive covers cash for transfers. Government of Madhya Pradesh also instituted Janani Express to provide vehicles for transfer of pregnant women to health institutions.
**Qualitative**								
14	Johnston 2003 [37]	Focus group discussions and In-depth interview	1999	Uttar Pradesh	Rural	Community	Men and women, women in reproductive age, post-abortion care providers	-
15	George 2007 [38]	In-depth interview	2004	Karnataka	Rural	Community	Pregnant women seeking delivery care	-
16	Vijayshree 2012 [39]	In-depth interview	2011	Karnataka	Rural	Not mentioned	Women seeking delivery care	-

^a^Both out and in- referrals

^b^No controls

^c^Abortions only

SC = Sub–Centre; PHC = Primary Health Centre; UHC = Urban Health Centre; ANC = Antenatal care; MO = Medical officer; ANM = Auxillary nurse midwife; VHW = Village health volunteer; TBA = Traditional birth attendant

#### Qualitative

Of the three studies (papers 14–16, Table 1), one conducted in-depth reviews and focus group discussions and the other two conducted only in-depth interviews among the pregnant women and/or care takers. One study was only about abortions (paper 14, Table 1). These studies were not scored on quality. However potential biases were identified.

#### Interventions

Five out of eight studies involving interventions focused on improving high risk identification during antenatal care (ANC) and referral by a medical officer (MO), ANMs, Village health volunteers (VHWs) or traditional birth attendants (TBAs) (papers 1–4 and 8, Table 1). Three studies also trained the ANMs, VHWs and TBAs for conducting safe deliveries, identification of complications and referrals (papers 3, 8 and 9, Table 1). In two studies MOs were trained for supervision of ANMs and VHWs. Only two studies focused on improving EmOC at primary level health institutions and referral to a higher level (papers 5 and 7, Table 1). However it should be noted that the institutions in these studies were primarily run by trained nurses and had an on-call medical officer or obstetrician available for opinion. In one study (paper 13, Table 1) a cash incentive transfer scheme, the Janani Suraksha Yojana (JSY) [42], implemented by the government covered the availability and cost aspects of referral transport. The state government where this study was based also had Janani Express Yojana [43], a system that transported pregnant women to health institutions.

#### Quality of included articles

Table 2 provides the scores of individual studies and summarises the potential biases as assessed by the reviewers. One paper (paper 1, Table 1) was found to be of poor quality. Although most of the studies intended to improve or report referrals, they did not report results completely. It was difficult to ascertain if the referrals were due to high-risk or complications.

**Table 2:** Quality scores (based on Strobe and Cochrane guidelines) and potential biases.

SNo	Author	Type of study	Scores based on STROBE/ CONSORT	Potential bias ^a^
**Out-referrals**				
1	Maitra 1995 [24]	Intervention- Prospective Cohort^b^	10/ 22 Poor quality	• Difficult to ascertain bias as the methods were not properly described. Results about timing and reasons for referral were also not clearly mentioned.
2	Hitesh 1996 [25]	Intervention–Prospective Cohort^b^	15/ 22 Medium quality	• Reporting bias: The details about when (during antenatal, intra-natal or post-natal period) the pregnant women were referred were not provided. Difficult to differentiate between referrals for high-risk or complications.
3	McCord 2001 [26]	Intervention–Prospective Cohort^b^	20/ 22 High quality	-
4	Barua 2003 [27]	Intervention–Prospective Cohort^b^	15/ 22 Medium quality	• Difficult to ascertain bias. Methods for baseline survey and facility survey not elaborated. Methods of surveillance and record keeping not mentioned.
5	Iyengar 2009 [28]	Intervention–Prospective Cohort^b^	19/ 22 High quality	• Reporting bias: The details (outcomes or referral) of the cases managed by the visiting obstetrician were not mentioned. Overall referral rates for the institution may be different.
6	More 2010 [29]	Cross-sectional (Study is a baseline before a trial.)	19/ 22 High quality	• Performance bias: Data collection was spread over two years during which some interventions had started. This could have led to contamination and influenced the outcomes. • Reporting bias: The details about when (during antenatal, intra-natal or post-natal period) the pregnant women experienced the reported complaints and were referred were not provided. Place of birth and pregnancy outcomes not mentioned. • Detection bias: Symptoms that are unrecognised, not thought to be serious or considered normal may lead to under reporting and limited care-seeking
7	David 2012 [30]	Intervention- Retrospective Cohort^b^	17/ 22 Medium quality	• Reporting bias: High risk pregnancies identified during ANC were referred to higher level health care and their deliveries were not attempted at the institution. The paper doesn’t report these numbers.
8	Alehagen 2012 [31]	Intervention–Prospective Cohort^b^	15/ 22 Medium quality	• Study was not planned as pre-post intervention study. • Difficult to ascertain bias as the methods of data collection are not elaborated. The baseline was assessed by a survey while the follow up data collection/ recording and frequency of recording is not described.
9	Pasha 2013 [32]	Cluster RCT^c^	20/ 25 High quality	• Performance bias: Intervention could not be completely implemented. • Blinding could not be done. • Reporting bias: Referral related process indicators were not reported.
**In-referrals**				
10	Biswas 2004 [33]	Cross-sectional	18/ 22 High quality	• Detection bias: The data were extracted from hospital records of past years. The quality of recording and diagnostic criteria may have varied over time. • Reporting bias: Reference period was not mentioned thus it was difficult to assess referral rates over time.
11	Kaul 2006 [34]	Cross-sectional	17/ 22 Medium quality	• Detection bias: The data was extracted from hospital records of past 4 years. The diagnostic criteria of post-partum haemorrhage for deliveries in the study hospital may vary from the referred cases whose deliveries were outside the study hospital. The later cases were also by selection more likely to be in moribund state due to time lost in travel and seeking care.
12	Banerjee 2012 [35]	Cross-sectional	19/ 22 High quality	• Detection bias: Symptoms of complications of abortions were self-reported and may vary in perception of relevance and seriousness.
13	Chaturvedi 2014 [36]	Cross-sectional	20/22 High quality	• Performance bias: Data collection was spread over one year but only 5 days in each of the 96 institutions. Five days of recruitment is a short period to comment on functioning and referral of an institution. Institution may try to perform better during the study period. • Reporting bias: The health institution were referred as primary, secondary and tertiary. It was not clear if primary level institutions were just delivery centres or BEmOC. Similarly, were the secondary level institutions providing all BEmOC functions or were CEmOC? This makes it difficult to assess the referral quality in review of obstetric emergencies.
**Qualitative**				
14	Johnston 2003 [37]	FGDs and In-depth interview	-	-
15	George 2007 [38]	In-depth interview	-	• Not planned as a scientific study. During a big study, 12 women seeking emergency obstetric care were impromptu followed and interviewed.
16	Vijayshree 2012 [39]	In-depth interview	-	• Difficult to ascertain bias. Source of sample and detail methods of data collection and analysis not mentioned.

^a^Taxonomy based on risk of Bias from Cochrane Handbook

^b^No controls

^c^Consort

### Out-referrals

Table 3 summarizes the findings on out-referrals. The relevant articles are discussed below.

**Table 3:** Summary findings of institution out-referrals for abortion, high-risk pregnancy, or complications in pregnancy/delivery.

Out-referrals for reasons:	Percentage of cases identified out of all pregnancies	Percentage of all pregnancies referred	Percentage compliance out of all referred	Second referral to higher institution
Abortion*	-	-	-	-
**Antenatal high-risk**				
*Subcentre/ community to PHC/ other institution*				
Maitra, 1995 [24]	**35%**	**6%**	**52.9%**	
Barua, 2003 [27]	35%-37%	35%-37%	-	
Aleghan, 2012 [31]	25%-52%	25%-52%	-	
**Complication or Emergency**				
*Subcentre/ community to PHC/ other institution (BEmOC or CEmOC)*				
Hitesh, 1996 [25]	-	-	**10.2%**	
Mc Cord, 2001 [26]	-	-	-	**4.7%**
Pasha, 2013 [32]	-	-	-	-
*Nurse run health centre or PHC (BEmOC) to First referral unit (FRU)/ CEmOC*				
Iyengar, 2009 [28] (All pregnancies in any phase of pregnancy)	**26.1%**	**19.7%**	**67%**	-
David, 2012 [30] (Low risk pregnancies for delivery care)	**36.3%**	**36.3%**	**68.6%**	-
Chaturvedi,2014 [36]		**14.3%**		
*Doctor run health centre (BEmOC) to higher institution (CEmOC)*				
More, 2011 [29]	-	**2%**	-	-
Chaturvedi, 2014 [36]		**7.5%**		

*cases of spontaneous abortions and post-induced abortion complication would have presented as complications in pregnancy

#### Referrals for abortion and post-abortion care

No article found.

#### Referrals for high-risk pregnancy

Three articles exclusively report on high-risk screening in pregnancy and out-referrals by community health staff or staff within a Subcentre [24,27,31]. All the three studies involved training ANMs for high-risk identification and referral to a higher level (PHC or Public Rural Hospital). The other 6 studies on out-referrals report on both high-risk cases and complications in pregnancy [25,26,28–30,32]. However these did not report the proportion referred for high risk in pregnancy. These are covered in the next section on complications in pregnancy.

**Proportion of out-referrals:** Maitra [24] reported that 4,522(35%) pregnancies in rural areas of 6 states were identified as being at high risk out of the total registered pregnancies (12,907). Only 786(17.6%) of the high risk pregnancies (i.e. 6% of all the pregnancies) were referred after intervention. However this paper scored poor on quality. Barua [27] also reported that after intervention, Subcentre ANMs found 35%-37% of registered pregnancies in rural areas in Maharashtra at high-risk and they referred them all to PHC medical officers. When anaemia and short-stature were excluded from these referrals then referrals to the PHC medical officer were only 18%. Alehgen [31] reported an increase from 25% cases identified and referred for high risk in 2006 to 52% in 2009 out of an estimated population of approximately 7,000 deliveries in a year in rural areas of Maharashtra.

In the Maitra and Barua studies, medical officers within the PHC further referred critical high-risk cases elsewhere for delivery (numbers and type of severity not reported) [24,27].

**Medical reasons for referrals:** Pre-eclampsia was detected in 11% and severe anaemia in 8% of all pregnant women in the study by Alehgen in 2012 [31].

**Institution-referral pathway and compliance:** During the baseline survey, Maitra, 1995 [24] and Barua, 2003 [27] noted that there were no referral records and no mechanisms whatsoever for identifying high-risk pregnancies before the intervention was implemented. These articles do not mention the type and quality of referral records that were maintained during the intervention.

Alehgen, 2012 [31] realized that the limited skills of available nurses and medical officers, and the high turn-over of staff, were limitations in establishing nurse-based ANC care. The study at baseline found that ANMs lacked equipment, skills and confidence to screen for high-risk, provide treatment, and refer appropriately. Similarly PHC medical officers lacked the skills and confidence to manage high-risk cases. As part of the intervention Subcentre ANMs and PHC medical officers were trained for their respective roles. Referrals were to be made to the next level of available public health institution. However there was no reporting of results, including whether any pregnant women were referred to a private health institution or if they complied with the choice of suggested referral institution. It is interesting to note that between 1995 and 2012 there was little improvement in high risk screening and referral [24,27,31].

In the study by Maitra in 1995, [24] most of the referrals made by the field workers were to a Subcentre. About half (52.9%) the women with high risk complied with referral.

#### Referrals for complications or emergency in pregnancy or puerperium

Six articles reported out-referrals for complications in pregnancy [25,26,28–30,32]. Two qualitative articles provide patient experiences of referrals for complications [38,39].

**Proportion of out-referrals:** Community health staff/ Subcentre to PHC/ BEmOC: In three studies [25,26,32] the intervention involved strengthening the skills of ANMs, TBAs or their equivalent in the community for ANC care, delivery care and referral. One of these studies [32] also provided training for home based life-saving skills to stabilise patients before referral. None of these studies report the proportion of high-risk or complication cases referred by these nurses to health institution.

Nurse run health centre (equivalent to PHC) / BEmOC to first referral unit (FRU) / CEmOC: One study in a rural area in Rajasthan by Iyengar, 2009 [28] and another in urban slums in Tamilnadu by David, 2012 [30] attempted to strengthen the skills of nurses at the health centres (equivalent to Primary health centre) to provide BEmOC services and referral under the mentorship of a visiting obstetrician [28] or physician [30]. The nurses in both studies did not induce labour in the absence of a doctor. In the first study 2,771 women presented in labour of whom 446(16.1%) were referred to FRU [28]. The second study based in urban slums referred all the high risk pregnancies to the next level of care and only attempted to assist 1,873 low risk pregnancies during the study period. Of these, 679(36.3%) were referred to a higher level for delivery and one post-partum hemorrhage case (out of 7) for treatment.

Mc-Cord in 2001 [26] reported that 20(4.7%) of 425 pregnant women from a rural study population in Maharashtra who attended the FRU were referred further onward for advanced specialist care. A study in urban slums in Maharashtra in 2011 [29] reported that less than 2% clients who sought care for any high-risk or complications at either public or private providers were referred to another health institution. The study also reports that as this urban population had access to range of health institutions, the community itself took efficient decisions on where to go based on their perceived severity of the symptoms and thus they may not have required further institution-referral.

Chaturvedi in 2014 [36] studied all the levels of health institutions which were assisting at least 10 deliveries in a month in 3 districts of Madhya Pradesh. Except PHCs, all other institutions had 24X7 medical officers available. The study reported that 5.9% of 1182 women seeking delivery care were referred out. The out-referral rate was highest from PHCs(14.3%) followed by CHCs(7.5%) and tertiary hospitals(0.8%). Half of the referrals from PHC were directly to tertiary hospitals, bypassing the CHCs.

**Medical reasons for referrals:** Among all the deliveries attempted or complications occurring at a nurse based rural health centre in Rajasthan, common reasons for referral were obstructed labour(25.1%), antepartum haemorrhage(16.2%), pregnancy induced hypertension(15.7%), severe anaemia(13.8%), complicated abortion(12.0%), post-partum haemorrhage(6.0%) and twin pregnancy(5.5%) [25]. Among low risk deliveries conducted at the nurse-based urban health centre, common reasons for referral were premature rupture of membranes(20%), failure to progress(15%), foetal distress (8.8%), pregnancy induced hypertension(10%), post-date pregnancies(6.2%) and grade-III meconium(6.3%) in early labour [30].

About two-fifths of the referrals from PHCs(43.1%) and three-fifths from CHCs (58.5%) in Madhya Pradesh were for prolonged labour and premature rupture of membranes followed by haemorrhage(10.3% in PHC and 6.6% in CHC) and eclampsia(3.4% in PHC and 6.6% in CHC). It is interesting to note that about 7% of referrals were due to facility dysfunction i.e. non-availability of staff, power or water [36].

**Institution-referral pathway and compliance:** In a 1996 study investigating the role of training, Hitesh reported that 206 women were issued a red referral card for high-risk or complications in 12 Subcentres [25]. Of these, only 21(10.2%) made any attempt to go to next level of health care. In the study in urban slums by David in 2012, 68.6% of referred women complied with referral [30], while in the study in rural areas by Iyengar in 2009, 74% of referred women complied [28]. Both the studies provided accompanying persons when required. Providers in the later study also arranged for transportation. Fixed rate private jeeps were available for transfer and for poor patients, nurses arranged transport for free or for subsidized rates.

Hitesh found that the most common (overlapping) reasons mentioned for non-compliance were cost(100%), lack of follow-up after reaching the institution(92.4%), TBA advised against it(92.4%), non-availability of transport(79.4%), previous bad experience(74.6%) and patients considering their symptoms normal(61.1%) [25]. Iyengar found that compliance was higher when complications occurred before the baby was born (78.7%), compared to those that occurred after delivery or abortion (57.1%) [28]. Patients with ante-partum haemorrhage and severe anaemia were difficult to convince about their need for referral. They tended to be apprehensive that relatives would be asked to donate blood [28].

#### Qualitative studies

The study by George et al in 2007 [38] reported case studies of 12 rural pregnant women in Karnataka who were seeking care for complications in pregnancy. These case studies highlighted that despite repeated visits to the public and private health care providers, patients did not obtain the required emergency obstetric care. The main reason identified was poor service delivery. The health systems had weak information systems, there was no continuity of care from antenatal to delivery and postpartum, peripheral health workers were unsupported and did not have the required skills, and there were haphazard referral systems and distorted accountability mechanisms when adverse events occurred [38]. Nine of the twelve women under study died despite seeking care in time.

Another study in Karnataka in 2012 of 10 users of EmOC services showed that these pregnant women received appropriate antenatal care but were not confident of where and when to get the EmOC services [39]. The designated FRU’s (CEmOC) had not been able to ensure 24 hour services every day of the week. All these women were living below the poverty line and belonged to scheduled castes or tribes. The six pregnant women with bad outcomes had gone through several referrals both in public and private health institutions. This was mostly due to non-availability of resources at the time of their visit. There was a time lapse of 10 hours to 32 hours to receive the required level of EmOC and there was a tendency to refer women at high-risk, or with complications, for the fear of facing maternal death audits and blame. The four women who had good outcomes received EmOC care due to interventions of caste-based organisations, local practitioners or concerned unions [39].

### In-referrals

#### Referrals for abortion and post-abortion care

Only one paper on in-referrals by Banarjee, 2012 [35] reported on referral related to abortion and complications of abortions.

**Proportion of in-referrals:** A total of 381 cases with complications after induced abortion and 405 after spontaneous abortion were interviewed. Eighty eight percent of the induced abortion group and 19.6% of the spontaneous abortion group had visited at least one institution before coming to the study hospitals [35].

Among the induced abortion group, 27(7%) came directly to the study hospitals for inducing an abortion, 10(5%) tried induction of abortion at home then came directly, 273(72%) visited one health institution, 59(12%) visited 2 institutions and 12(3%) visited 3 institutions before coming to one of the study hospitals. Among the spontaneous abortion group 327(80.4%) came directly to study hospitals and the rest 78(19.6%) visited one health institution before coming to the study hospitals.

**Medical reasons for referrals:** The paper mentions the self-reported symptoms but nothing about specific reasons for institution-referral.

**Institution-referral pathway and compliance:** Most of the women were not aware if the health providers they visited were qualified or not [35]. A qualitative paper by Johnston, 2003 [37] interviewing post-abortion care providers in study villages in rural Uttar Pradesh revealed that pregnant women consulted the local village-level providers for abortion care rather than going to the nearest health institution. Village-level providers were all un-qualified practitioners, however the pregnant women thought they were qualified. These village-level providers tended to provide abortion and post-abortion care rather than refer to more appropriate providers. If the case was critical they would refer the case to the nearest town, however no specific health institution was mentioned.

#### Referrals for High-risk in pregnancy

No article found.

#### Referrals for complications or emergency in pregnancy or puerperium

Three articles reported in-referrals for complications in pregnancy [33,34,36].

**Proportion of in-referrals:** Biswas in 2004[30] reported that on an average 5–10% of all in-patients at FRUs in West Bengal were in-referrals from peripheral health institutions (estimate as told by the head of institutions). In a tertiary hospital in Haryana, Kaul in 2006 [34] reported that 90(0.6%) of 13,907 deliveries developed post-partum hamorrhage and another 88 PPH cases were referred in after having delivered elsewhere. Nineteen women(10.7%) suffered “near-miss” morbidity (5 in hospital delivery and 14 referred cases).

Chaturvedi in 2014 [36] found that 111(9.4%) of 1,182 women seeking delivery care were referred in from other institutions. None of the cases referred in by other institutions required to be referred out again. The proportion of in-referrals was highest in government tertiary institutions(21.2%) followed by private hospitals(16.1%), both of which were working as CEmOCs. CHCs which were working as BEmOCs received only 1.6% in-referrals. It is important to note that the average number of women in labour per institution was 121 for tertiary institutions, 21 for CHCs, 6 for private hospitals and 4 for PHCs over a five day period [36].

**Medical reasons for referrals:** Not specified.

**Institution-referral pathway and compliance:** Kaul found that 54(61.3%) of 88 referred PPH cases were transferred in more than 6 hours after delivery elsewhere. All 14 near-miss cases in the referred group reached the tertiary hospital more than 6 hours following delivery [34]. In Madhya Pradesh, 63% in-referrals used a Janani Express vehicle. The average inter-institution travel time was 1.25 hours [36]. About three-quarters (72%) in-referred cases had a referral slip however they mostly did not contain the reasons for referral and the treatment provided before referral [36]. The superintendents of FRUs [34] stated that there were no records for in-referrals at any FRU and only 2 out of 12 FRU studied had some records for out-referrals. The in-referred cases did not carry referral notes, and there were no mechanisms for providing feedback to referring units.

A recent study in 2014 reported that 97% of referrals were before delivery, and 60% were admitted at the first institution before referral. Most of the first referrals were received at government tertiary care centers(73%), followed by private hospitals(15.3%) and CHCs(11.7%). The former two worked as CEmOCs, and the CHCs were mostly BEmOCs. Most of the first referrals at tertiary care centres were received from CHCs(65.4%) and PHCs(24.7%). Most of the first referrals at private care centres were from CHCs(70.6%) and remaining were from other private hospitals. None of the referrals from PHCs were received in private hospital. There were 13 second referrals, received at government tertiary care centres and private hospitals [36].

Table 4 summarises the problem issues in referral of obstetric cases.

**Table 4:** Problem issues identified in institution-referrals for obstetric high-risk or complications.

**Obstetric care and the proportion of referrals**
1. High proportion of referrals from the peripheral health institutions.
2. Low skills and confidence of peripheral staff in identifying high-risk and complications, and providing stabilising care.
**Classification of high risk pregnancy or complications in pregnancy**
3. Confusion in the clinical criteria for referral: Some high-risk cases can be managed at BEmOC and may not need referral. Only the complication cases need to be referred. Clear definitions can help decide for appropriate referrals and avoid unnecessary referrals.
4. No standard guidelines for the management of high-risk conditions and complications at BEmOC. This could avoid unnecessary referrals.
5. Low confidence of nursing staff at delivery centres and PHCs to manage high-risk pregnancies and to induce labour despite SBA trainings, established referral linkages and transportation services.
**Reaching appropriate referral facility**
6. Bypassing CHCs: PHCs prefer to refer straight to district level secondary and tertiary care centres. This may be due to lack of information at Subcentres and PHCs about services available at mid-level institutions (CHC).
7. Non- uniform standards and availability of care despite defining an institution as PHC or CHC or BEmOC or CEmOC.
8. No transport interventions specifically for referrals between institutions.
**Quality of referral**
9. No emphasis on the quality of referral advice, referral notes and keeping referral records.
10. No formal communication and transportation arrangements between the institutions.
11. No audit on quality of antenatal and delivery care including referral from the peripheral centres.
12. Poor compliance: Need for complications awareness and readiness in the community, and emphasis on referral counselling.

## Discussion

### Level of obstetric care and proportion of referrals

This review suggests that about one-third to one-half of pregnancies in rural populations are assessed as high risk and are referred from a Subcentre to a PHC or CHC for further antenatal check-up and delivery care (Table 3). Almost half of these are anaemia or short stature cases which add large numbers to the high risk obstetric population in India. Simultaneously, low risk pregnant women delivering at Nurse run PHCs or Urban centres, which are capable of providing BEmOC care (except induction of labour), refer up to one-fifth to one-third of cases to a higher level institution. These findings suggest that about one-half to two-thirds of all pregnant women attending lower level health institutions are likely to be referred during pregnancy or delivery (Table 3). Studies in Africa suggested similar proportions. If protocols for antenatal high-risk identification and referral, along with referral for complications, are followed then 35–50% of pregnant women in Africa will need to be referred from peripheral institutions to the next level of care [44].

Two studies also found that, ANMs, Nurses and even MOs were not confident and did not have skills to provide EmOC and referral care [31,45]. The under-confident and unskilled health staff are likely to refer higher proportions of pregnant women on the slightest of indication of high risk or complication. The selected intervention studies in this review did not have pre-intervention proportions and comparison groups to ascertain above hypothesis.

### Referral for high risk in pregnancy or complication in pregnancy

A study conducted in Tanzania in 2009 reported that 28% of women registered for ANC at peripheral health centre were referred to higher level hospitals. Out of the referred patients, 70% were referred due to demographic risks, 12% due to obstetric historical risks, 12% with prenatal complications and 5.5% with delivery and immediate postnatal complications. Only half of these referred women complied, and these were mainly women with obstetric historical risks and any complications [46]. The proportion of referrals in this study are lower than nurse-run centres in our review, probably because the centre in the Tanzanian study was run by a clinical doctor. A clinical doctor or a MO is likely to be more capable of managing obstetric high risk and complications as compared to the nurses, thus reducing the number of referrals to next level. One study in our review [36] suggested that 14.3% women were referred from a PHC and about 7.5% from CHC for delivery complications alone compared to 5.5% in the Tanzanian study. The commonest causes for referral were prolonged labour and rupture of membranes which could have–in theory- been managed at the referring institutions [36]. This suggests that there is tendency for over and unnecessary referral from peripheral institutions in the government sector.

We also observed that definitions of high risk and complications and referral indications were not uniform in the studies. The studies prior to 2000 focused primarily on high risk screening during antenatal care by ANM, VHWs or TBAs while in the later studies the focus was also on delivery services, identification of historic obstetric high-risks and complications, and referral from primary care institutions. It appears that antenatal high-risk identification and referral still continues although the Safe Motherhood strategies now emphasize providing basic natal care to all with early identification of obstetric complications and providing referral to appropriate EmOC care [47]. Decisions about referrals are often very complicated and confusing in the absence of guidelines.

Studies show that high risk prediction may not necessarily mean that the woman will have complication and many women identified as being at risk go on to have normal deliveries [48,49]. Jahn and De Brouwere identified a core set of indications for referral which would produce referral rates of 6% to 10% and reduce a lot of un-necessary high-risk referrals. These include mainly previous caesarean section, breech presentation, transverse lie, multiple gestation, hypertension, and severe anaemia [7]. One of the community-based studies in the review found that only 14.4% of all deliveries had any complications [26], and another study by Bang et al found that only 17.7% had any complications [50]. This suggests that if all the deliveries were to be managed at a functional BEmOC (capable of managing high risk) then there may not be high proportion of referrals to CEmOC.

### Reaching an appropriate referral institution for high risk, complication or emergency

Findings of qualitative studies in the review suggest that referrals are haphazard and a pregnant woman at high risk or with complications did not get the required EmOC and had to go through several referrals before reaching the appropriate institution. The high proportion of referrals and the experiences faced during referral are probably a reason why pregnant women in India choose to deliver at private institutions or go directly to higher level government institutions to avoid the transfers [31,45].

A study by Chaturvedi in 2014 suggests that referrals from PHC were justified, however 69% were directly to more trusted tertiary care bypassing the CHC. About half the referrals from CHCs could have been managed at the CHC. Bypassing CHCs by the PHC, and unjustified referrals from CHC, point towards distrust and lack of confidence in CHCs for the management of complications. Interestingly, none of the in-referrals in any of the institutions in this study required to be referred further. Only 7.2% referrals had either two or three referrals [36].

Although many interventions have been implemented in India in the last decade for the transportation of pregnant women there are no interventions specifically for referrals between institutions [42,43]. Most PHCs and CHCs do not have their own ambulances and rely on services like ‘108’ ambulances or other public private transports for transfers [51].

Assessment of CEmOCs in various states of India have shown that the number of complicated deliveries handled at referral institutions is far below the estimated need of around 15% of all pregnancies and 100% of all complications [33,52,53]. Despite of high proportion of referrals from lower institutions the referrals received at higher institutions for complications is highly inadequate. This may be due to poor compliance to referral or referrals are mostly for non-complicated high risk pregnancies or normal deliveries. A study in Tanzania suggested that only 1.2% of referrals to a referral institution were for any complication or emergency. Amongst the rest, 18% were high risk cases referred during the antenatal care [54].

### Quality of referral

Studies in our review also reported that there were no referral records maintained and no proper referral documents provided to the pregnant women at the time of referral. Only in one study 73% of referrals were provided referral slips but they did not provide any information about clinical manifestations or treatment [36]. Studies in the review found that the complication cases were not adequately stabilised nor were they given first-line treatment before referral, and a large proportion did not comply with the referral at all. A few chose to go to their preferred institution. This may be due to poor communication and counselling skills of the sender, high cost, non-availability of transport and distrust in the referral institution. Compliance was better in the presence of an accompanier from the referring institution or where the nurse arranged for the transport and communicated about the case to the higher level institution [5].

India has a 108 free ambulance service in most parts of the country which is the mainstay for transfer from home and between institutions for pregnant women [55]. However a study in Gujarat on referral systems and transportation revealed that the focus of the system was more on the number of ambulances and drivers, and less on the number of referrals transported [21]. In Madhya Pradesh 63% of the referred cases used a Janani Express vehicle and average travel time was 1.25 hours between institutions. The study identified that factors contributing to poor quality of referral and delay in getting care were less about the availability of transport, due to higher utilization of schemes like JSY and Janani Express vehicle, than inadequacies of the staff in identification of complications, referral and communication with the nearest appropriate institution and provision of pre-transfer first-line treatment. The study also identified delays in receiving care at the referral institution [36].

There was overall lack of monitoring of the referral system and accountability to patients. Murray et.al [56] recommends supervision and increasing accountability of care providers. Strand et.al [57] and Konganyuy et.al [58] suggest audits of referrals for obstetric emergencies to improve referral systems for obstetric care and prevent delays.

**Fig 3:**
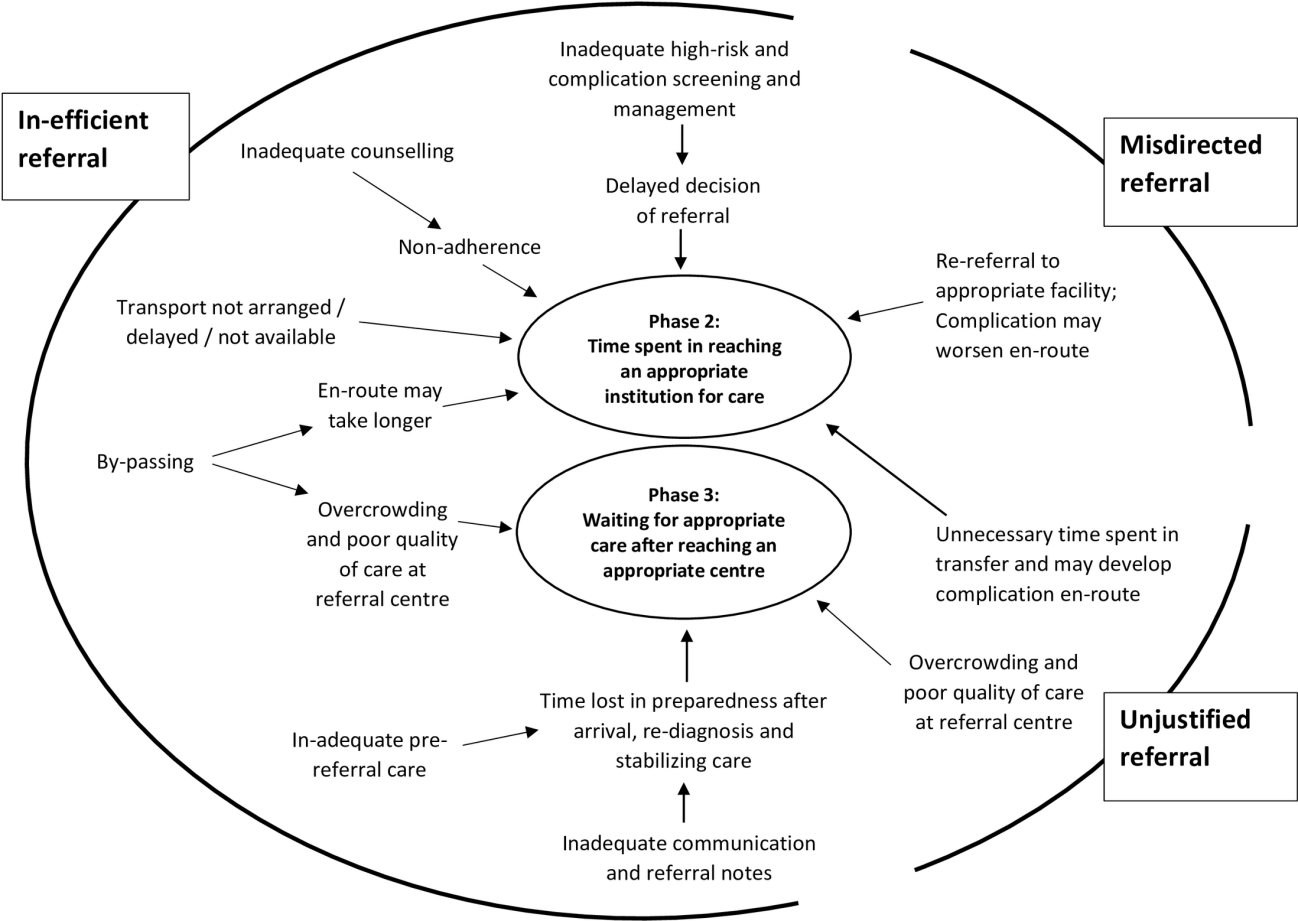
Inappropriate institution-referrals and contribution to delays in access to emergency obstetric care.

Three phases of delay regarding access to appropriate emergency obstetric care have been identified: Phase 1 delay caused by time spent in decision making; phase 2 delay resulting from time spent reaching an appropriate institution for care; and phase 3 delay caused by waiting for appropriate care after reaching an appropriate centre [59]. Inefficient institution-referral systems contribute mostly to phase 2 delays but may also contribute to phase 3 delays due to non-readiness of the hospital because of poor communication about the arrival. Misdirected referral may also lead to phase 2 delays by sending the patient to an institution which is incapable of managing the referred condition. This may even increase the severity of complication in due course.[10] On the other hand unjustified referrals may lead to underutilization of some centres and overcrowding at others [54]. Fig 3 describes the components of inappropriate institution-referrals and contribution to delays in obstetric care as understood from the review. Further research is required to understand the determinants of each of these components in Indian context.

A critical review on maternity referral systems, mainly institution-referrals, in developing countries found that there was considerable disparity between the hierarchical referral pyramid found in policy documents and the realities for women to access maternity care in many urban and rural settings [56]. Successful implementation of each referral system needs: a referral strategy informed by the assessment of population needs and health system capabilities, an adequately equipped referral institution, specific referral protocols, active collaboration between referral levels and other sectors, established communication and transport arrangements, affordable service costs, supervision and accountability for quality of care, the capacity to monitor effectiveness and policy support [56].

### Strengths and Limitations

The systematic review is first of its kind to summarise evidence on referrals across different levels of care and for different indications in obstetric care in India. Restricting the review to India helped to understand referral criteria and pathways in the context of Indian health systems. The review emphasizes the need for development of clear referral protocols, and for the resources to implement them. The findings from the review will help the programmers to have an estimate of referrals and resources required, and also to identify future intervention research.

A few studies identified in this literature search were not included as they did not clearly mention if the participants were self-referrals or institution referrals. A few others did not mention results appropriately on proportions of referrals and causes of referrals. These studies, if reported well, could have added more evidence. Among the selected studies on out-referrals, the outcomes of pregnancy in terms of maternal and newborn morbidity and mortality and modes of transport and costs were not well reported. Thus we could not link the referrals with these variables. Most of the studies on out-referrals used interventions to improve referrals and did not report pre-intervention referral proportions, or have controls. It was thus difficult to combine results of the intervention and cross-sectional studies.

## Conclusions

The proportion of institution-referrals was high. Referrals are a huge burden on the Indian health system, especially regarding transportation and management at higher institutions. Along with this, poor referrals may contribute to phase 2 and phase 3 delays. The high proportion of institution-referrals and pathways of referrals in India point towards a) the inability of primary health centres to provide basic delivery care and BEmOC services, b) inadequate pre-referral stabilizing care, c) a tendency for unjustified referrals to higher institutions, d) bypassing the CHCs as first referral choice, e) inadequate referral communication and record maintenance, and f) absence of standard guidelines for referral, facilities and monitoring of referrals for obstetric care.

Studies are required to assess the referral practices and problems faced by staff at lower level health institutions to decide when, where and how to refer the pregnant women. Strategies need to be developed a) to provide supervision and support to nurses for better BEmOC and referral, b) to standardize treatment and referral protocols and pathways, and c) monitor the quality of obstetric care and referrals from lower level health institutions and receiving these referrals at higher institution.

## Supporting Information

S1 PRISMA Checklist Prisma 2009 Checklist. (DOC)

S1 Text Search strategy for Referrals between public sector institutes for women with obstetric high risk, complications, or emergencies in India–A Systematic review. (DOCX)

S2 Text (DOCX)
