# Supplementary material for: Referrals between Public Sector Health Institutions for Women with Obstetric High Risk, Complications, or Emergencies in India – A Systematic Review
Source: PLoS One. 2016 Aug 3;11(8):e0159793. doi: 10.1371/journal.pone.0159793 (PMC4972360; doi:10.1371/journal.pone.0159793)
Supplement: S1 Text — (DOCX) [file pone.0159793.s002.docx]

**Search strategy for- Referrals between public sector institutes for women with obstetric high risk, complications, or emergencies in India – A Systematic review**

1. "referral and consultation"/ or gatekeeping/ or physician self-referral/ or remote consultation/ or secondary care/ or tertiary healthcare/

2. (referral adj2 pathway*).mp. [mp=title, abstract, original title, name of substance word, subject heading word, keyword heading word, protocol supplementary concept word, rare disease supplementary concept word, unique identifier]

3. remote consultation/

4. telemedicine/

5. exp Transportation/

6. exp Transportation of Patients/

7. patient transfer/

8. exp transfer/

9. time factors/

10. communication/

11. telecommunications/

12. exp telephone/

13. (phone* or telephone* or cellphone* or mobile phone* or radio*).mp. [mp=title, abstract, original title, name of substance word, subject heading word, keyword heading word, protocol supplementary concept word, rare disease supplementary concept word, unique identifier]

14. interdisciplinary communication/

15. exp Emergency Medical Service Communication Systems/

16. emergency medical services/ or emergency service, hospital/

17. emergency treatment/

18. Emergencies/

19. triage/

20. First Aid/

21. exp hospitalization/

22. Maternal-Child Health Centers/

23. delivery of healthcare/

24. Health Services Accessibility/

25. health education/ or consumer health-information/

26. Ambulances/ or Air Ambulances/

27. (emergency adj1 (vehicle* or transport* or van* or car* or cycle* or bicycle* or ambulance*)).mp. [mp=title, abstract, original title, name of substance word, subject heading word, keyword heading word, protocol supplementary concept word, rare disease supplementary concept word, unique identifier]

28. (emergenc* adj2 (respons* or referral*)).mp. [mp=title, abstract, original title, name of substance word, subject heading word, keyword heading word, protocol supplementary concept word, rare disease supplementary concept word, unique identifier]

29. ((health or communit*) adj5 (work* or participant* or profession* or educat* or fund* or service*)).mp. [mp=title, abstract, original title, name of substance word, subject heading word, keyword heading word, protocol supplementary concept word, rare disease supplementary concept word, unique identifier]

30. (refer or referral or transfer* or transport* or communication* or emergenc* or ambulance).mp. [mp=title, abstract, original title, name of substance word, subject heading word, keyword heading word, protocol supplementary concept word, rare disease supplementary concept word, unique identifier]

31. ((train* or educat*) adj5 (matern* or health* or professional or doctor* or midwive* or midwife* or nurs*)).mp. [mp=title, abstract, original title, name of substance word, subject heading word, keyword heading word, protocol supplementary concept word, rare disease supplementary concept word, unique identifier]

32. (ANM* or Midwife* or Nurse* or Skilled birth attendant* or Doctor* or Obstetr* or TBA*).mp. [mp=title, abstract, original title, name of substance word, subject heading word, keyword heading word, protocol supplementary concept word, rare disease supplementary concept word, unique identifier]

33. (Hospital or health center).mp. [mp=title, abstract, original title, name of substance word, subject heading word, keyword heading word, protocol supplementary concept word, rare disease supplementary concept word, unique identifier]

34. emergency service, hospital/ or trauma centers/

35. or/1-34

36. pregnancy/ or labo?r, obstetric/ or exp pregnancy outcome/

37. exp Pregnancy Complications/

38. delivery, obstetric/ or extraction, obstetrical/ or labor, induced/

39. Obstetrics/

40. exp Obstetric Labor Complications/

41. maternal health services/ or perinatal care/ or postnatal care/ or prenatal care/

42. obstetric care/

43. Maternal Mortality/

44. (ante-natal or antenatal or pre-natal or prenatal or natal or ante-partum or antepartum or post-natal or postnatal or post-partum or postpartum).mp. [mp=title, abstract, original title, name of substance word, subject heading word, keyword heading word, protocol supplementary concept word, rare disease supplementary concept word, unique identifier]

45. (peri-natal or perinatal).mp. [mp=title, abstract, original title, name of substance word, subject heading word, keyword heading word, protocol supplementary concept word, rare disease supplementary concept word, unique identifier]

46. (labo?r or delivery or birth or childbirth).mp. [mp=title, abstract, original title, name of substance word, subject heading word, keyword heading word, protocol supplementary concept word, rare disease supplementary concept word, unique identifier]

47. pregnan*.mp. [mp=title, abstract, original title, name of substance word, subject heading word, keyword heading word, protocol supplementary concept word, rare disease supplementary concept word, unique identifier]

48. matern*.mp. [mp=title, abstract, original title, name of substance word, subject heading word, keyword heading word, protocol supplementary concept word, rare disease supplementary concept word, unique identifier]

49. (maternal adj3 (morbidity or mortality or outcome*)).mp. [mp=title, abstract, original title, name of substance word, subject heading word, keyword heading word, protocol supplementary concept word, rare disease supplementary concept word, unique identifier]

50. infant/ or infant, newborn/

51. neonat*.mp.

52. (neonat* adj3 (morbidity or mortality or outcome or infections or illness)).mp. [mp=title, abstract, original title, name of substance word, subject heading word, keyword heading word, protocol supplementary concept word, rare disease supplementary concept word, unique identifier]

53. obstructed labo?r.mp. [mp=title, abstract, original title, name of substance word, subject heading word, keyword heading word, protocol supplementary concept word, rare disease supplementary concept word, unique identifier]

54. (eclampsia or pre-eclampsia).mp. [mp=title, abstract, original title, name of substance word, subject heading word, keyword heading word, protocol supplementary concept word, rare disease supplementary concept word, unique identifier]

55. ((obstetric or postpartum or post-partum) adj3 h?emorrhage).mp. [mp=title, abstract, original title, name of substance word, subject heading word, keyword heading word, protocol supplementary concept word, rare disease supplementary concept word, unique identifier]

56. ((genital or urin*) adj3 infect*).mp. [mp=title, abstract, original title, name of substance word, subject heading word, keyword heading word, protocol supplementary concept word, rare disease supplementary concept word, unique identifier]

57. (ruptur* adj4 (uterus or uterine)).mp. [mp=title, abstract, original title, name of substance word, subject heading word, keyword heading word, protocol supplementary concept word, rare disease supplementary concept word, unique identifier]

58. ((prolonged or obstructed) adj2 (labo?r or birth or delivery)).mp. [mp=title, abstract, original title, name of substance word, subject heading word, keyword heading word, protocol supplementary concept word, rare disease supplementary concept word, unique identifier]

59. ((postpartum or post-partum) adj1 (sepsis or septicemia or fever)).mp. [mp=title, abstract, original title, name of substance word, subject heading word, keyword heading word, protocol supplementary concept word, rare disease supplementary concept word, unique identifier]

60. ((thirdstage or third-stage) adj1 labo?r).mp. [mp=title, abstract, original title, name of substance word, subject heading word, keyword heading word, protocol supplementary concept word, rare disease supplementary concept word, unique identifier]

61. (BEmOC or EmOC or CEmOC).mp. [mp=title, abstract, original title, name of substance word, subject heading word, keyword heading word, protocol supplementary concept word, rare disease supplementary concept word, unique identifier]

62. ((highrisk* or high-risk*) adj2 (pregnan* or obstetric* or gestation* or delivery or labo?r or birth or antenatal or ante-natal or prinatal or peri-natal or postnatal or post-natal)).mp. [mp=title, abstract, original title, name of substance word, subject heading word, keyword heading word, protocol supplementary concept word, rare disease supplementary concept word, unique identifier]

63. (matern* adj2 (hospital or center or health center)).mp. [mp=title, abstract, original title, name of substance word, subject heading word, keyword heading word, protocol supplementary concept word, rare disease supplementary concept word, unique identifier]

64. (emergenc* adj2 obstetric*).mp. [mp=title, abstract, original title, name of substance word, subject heading word, keyword heading word, protocol supplementary concept word, rare disease supplementary concept word, unique identifier]

65. (obstetric adj3 care*).mp. [mp=title, abstract, original title, name of substance word, subject heading word, keyword heading word, protocol supplementary concept word, rare disease supplementary concept word, unique identifier]

66. or/36-65

67. India.mp. or India/

68. 35 and 66 and 67

69. limit 68 to (human and english language and yr="1991 -Current")
