# Supplementary material for: Referrals between Public Sector Health Institutions for Women with Obstetric High Risk, Complications, or Emergencies in India – A Systematic Review
Source: PLoS One. 2016 Aug 3;11(8):e0159793. doi: 10.1371/journal.pone.0159793 (PMC4972360; doi:10.1371/journal.pone.0159793)
Supplement: S2 Text — (DOCX) [file pone.0159793.s003.docx]

List of 215 articles and reports whose full texts were read but were excluded.

Reason of exclusion **1. No mention of obstetric referral**

**2. No description of institution-referral proportions and pathways**

| Si.No. | **Paper** | **Reason** |
| --- | --- | --- |
| 1 | S. Prinja, G. Jeet, M. Kaur, A. K. Aggarwal, N. Manchanda and R. Kumar. Impact of referral transport system on institutional deliveries in Haryana, India. Indian J Med Res. 2014;139:883-891 | 2 |
| 2 | P. B. Patel, M. P. Rupani and S. S. Patel. Antenatal care registration and predicting factors of late registration among pregnant women. Tropical Doctor. 2013;43(1): 9-12. | 1 |
| 3 | P. K. Mony, J. Krishnamurthy, A. Thomas, K. Sankar, B. M. Ramesh, S. Moses, J. Blanchard and L. Avery. Availability and distribution of emergency obstetric care services in Karnataka State, South India: access and equity considerations. PLoS ONE. 2013;8:5:e64126. | 2 |
| 4 | K. P. Gill, P. Devgun, S. L. Mahajan and A. Chopra. Delivery practices and their determinants in urban slums of Amritsar City, Punjab, India. Indian Journal of Public Health Research and Development. 2013;4(3):133-137. | 1 |
| 5 | S. Sinha, R. P. Upadhyay, J. P. Tripathy and B. K. Patro. Does utilization of antenatal care result in an institutional delivery? Findings of a record-based study in urban Chandigarh. Journal of Tropical Pediatrics. 2013;59(3):220-2. | 1 |
| 6 | M. Parashar, S. V. Singh, J. Kishore, A. Kumar and M. Bhardwaj. Effect of community-based behavior change communication on delivery and newborn health care practices in a resettlement colony of Delhi. Indian Journal of Community Medicine. 2013;38(1):42-48. | 1 |
| 7 | S. Amudhan, S. K. Rai, C. S. Pandav, A. Krishnan and K. Mani. Effectiveness of demand and supply side interventions in promoting institutional deliveries - a quasi-experimental trial from rural North India. International Journal of Epidemiology. 2013;42(3):769-780. | 1 |
| 8 | P. B. Ramachandra Bhat, M. H. Navada, S. V. Rao and G. Nagarathna. Evaluation of obstetric admissions to intensive care unit of a tertiary referral center in coastal India. Indian Journal of Critical Care Medicine. 2013;17(1):34-37. | 2 |
| 9 | M. Bakliwal. Evaluation of the referred cases from rural areas for a period of 5 years to the obstetric department of a Teaching Hospital. Der Pharmacia Lettre. 2013:5(1);80-82. | 2 |
| 10 | P. Pahwa and A. Sood. Existing practices and barriers to access of MCH services – a case study of residential urban slums of district Mohali, Punjab, India. Global Journal of Medicine and Public Health. 2013;2:4 | 1 |
| 11 | A. Subramaniyan, S. Sarkar, G. Roy and S. Lakshminarayanan. Experiences of HIV positive mothers from rural South India during intra-natal period. Journal of Clinical and Diagnostic Research. 2013;7(10):2203-2206. | 1 |
| 12 | M. P. Roy, U. Mohan, S. K. Singh, V. K. Singh and A. K. Srivastava. Factors associated with the preference for delivery at the government hospitals in rural areas of Lucknow district in Uttar Pradesh. Indian journal of public health. 2013;57(4):268-271. | 1 |
| 13 | B. Randive, V. Diwan and A. De Costa. India’s conditional cash transfer programme (the JSY) to promote institutional birth: Is there an association between Institutional birth proportion and maternal mortality? . PLOS one. 2013;8(6): e67452 | 2 |
| 14 | H. Konar and A. B. Chakraborty. Maternal mortality: A FOGSI study (Based on institutional data). Journal of Obstetrics and Gynecology of India. 2013;63(2):88-95. | 1 |
| 15 | S. S. Raj, D. Maine, P. K. Sahoo, S. Manthri and K. Chauhan. Meeting the community halfway to reduce maternal deaths? Evidence from a community-based maternal death review in Uttar Pradesh, India. Global Health: Science and Practice. 2013;1(1):84-96. | 2 |
| 16 | H. Siddalingappa, M. M. R. Narayana, P. Kulkarni and N. C. Ashok. Prevalence and factors influencing perinatal mortality in rural Mysore, India. Journal of Clinical and Diagnostic Research. 2013;7(12):2796-2799. | 2 |
| 17 | Leema, R. Sehgal, S. Mittal, Shobha, V. L. Jindal, A. Kumar and Lalit. Psychological assessment of primiparous women during the ante & postnatal period a longitudinal study of 3 years. Indian Journal of Public Health Research and Development. 2013;4(3):170-175. | 1 |
| 18 | A. Rammohan, K. Iqbal and N. Awofeso. Reducing Neonatal Mortality in India: Critical Role of Access to Emergency Obstetric Care. PLoS ONE. 2013;8:3. | 2 |
| 19 | S. R. Shrivastava and P. S. Bobhate. Study to assess utilization of antenatal and intranatal services amongst women in an urban slum of Mumbai. TAF Preventive Medicine Bulletin. 2013;12(2):157-164. | 1 |
| 20 | M. Hsiao, S. K. Morris, A. Malhotra, W. Suraweera, and Prabhat Jha. Time-critical mortality conditions in low-income and middle-income countries. Lancet. 2013;381:993-4; | 1 |
| 21 | R. P. Upadhyay, S. K. Rai and A. Krishnan. Using three delays model to understand the social factors responsible for neonatal deaths in rural Haryana, India. Journal of Tropical Pediatrics. 2013;59(2):100-105. | 1 |
| 22 | A. Pandey, V. Das, A. Agarwal and S. Agarwal. A retrospective study of "near miss" obstetric events and maternal deaths in a tertiary health care center of North India. International Journal of Gynecology and Obstetrics. 2012;119:S449-S450. | 1 |
| 23 | A. Shukla and T. Bhatnagar. Accredited social health activists and pregnancy-related services in Uttarakhand, India. BMC Proceedings. 2012;6. | 1 |
| 24 | Saji S. Gopalan and Durairaj Varatharajan. Addressing maternal healthcare through demand side financial incentives: experience of Janani Suraksha Yojana program in India. BMC Health Services Research. 2012;12:319 | 1 |
| 25 | K. Sidney, A. de Costa, V. Diwan, D. V. Mavalankar and H. Smith. An evaluation of two large scale demand side financing programs for maternal health in India: the MATIND study protocol. BMC Public Health. 2012;12:699. | 2 |
| 26 | B. S. Sri, N. Sarojini and R. Khanna. An investigation of maternal deaths following public protests in a tribal district of Madhya Pradesh, central India. Reproductive Health Matters. 2012;20:11-20. | 1 |
| 27 | A. Chauhan. Antenatal care among currently married women in Rajasthan, India. Asian Pacific Journal of Tropical Disease. 2012;2(SUPPL2):S617-S623. | 1 |
| 28 | M. Viegas Andrade, K. Noronha, A. Singh, C. G. Rodrigues and S. S. Padmadas. Antenatal care use in Brazil and India: Scale, outreach and socioeconomic inequality. Health and Place. 2012;18(5):942-950. | 1 |
| 29 | T. K. Panja, D. K. Mukhopadhyay, N. Sinha, A. B. Saren, A. Sinhababu and A. B. Biswas. Are institutional deliveries promoted by Janani Suraksha Yojana in a district of West Bengal, India?. Indian journal of public health. 2012;56(1): 69-72. | 1 |
| 30 | P. C. Adamson, K. Krupp, B. Niranjankumar, A. H. Freeman, M. Khan and P. Madhivanan. Are marginalized women being left behind? A population-based study of institutional deliveries in Karnataka, India. BMC public health. 2012;12:30. | 1 |
| 31 | L. Singh, R. K. Rai and P. K. Singh. Assessing the utilization of maternal and child health care among married adolescent women: evidence from India. Journal of biosocial science. 2012;44(1):1-26. | 1 |
| 32 | S. Awasthi, M. Chaturvedi, D. Nandan, S. K. Jha and A. K. Mehrotra. Assessment of quality maternity care in Urban slums of district Agra: Population based study. Indian Journal of Public Health Research and Development. 2012;3(4):186-190. | 1 |
| 33 | N. Seward, D. Osrin, L. Li, A. Costello, A. M. Pulkki-Brannstrom, T. A. Houweling, J. Morrison, N. Nair, P. Tripathy, K. Azad, D. Manandhar and A. Prost. Association between clean delivery kit use, clean delivery practices, and neonatal survival: pooled analysis of data from three sites in South Asia. PLoS medicine. 2012;9:2. e1001180. | 1 |
| 34 | A. K. Chaudhary, C. Asha, S. C. Tiwari and R. Dwivedi. Can community-based, low-cost antenatal care in the third trimester of pregnancy reduce the incidence of low birth weight newborns? Journal of Obstetrics and Gynecology of India. 2012;62(3):286-290. | 1 |
| 35 | V. Kumar, A. Kumar, V. Das, N. M. Srivastava, A. H. Baqui, M. Santosham, et al. Community-driven impact of a newborn-focused behavioral intervention on maternal health in Shivgarh, India. International Journal of Gynecology and Obstetrics. 2012;117:48-55 | 2 |
| 36 | B. Randive, S. Chaturvedi and N. Mistry. Contracting in specialists for emergency obstetric care- does it work in rural India? BMC health services research. 2012;12:485. | 2 |
| 37 | P. K. Singh, R. K. Rai, M. Alagarajan and L. Singh. Determinants of maternity care services utilization among married adolescents in rural India. PloS one. 2012;7:2. e31666. | 1 |
| 38 | K. P. Allagh and A. Thippaiah. Developing training modules for nurses in safe motherhood. Indian Journal of Public Health Research and Development. 2012;3(2):93-96. | 1 |
| 39 | S. S. Goudar, S. M. Dhaded, E. M. McClure, R. J. Derman, V. D. Patil, N. S. Mahantshetti, R. M. Bellad, B. Kodkany, J. Moore, L. L. Wright, W. A. Carlo and I. study. ENC training reduces perinatal mortality in Karnataka, India. Journal of Maternal-Fetal & Neonatal Medicine. 2012;25(6):568-74. | 1 |
| 40 | A. J. Barros, C. Ronsmans, H. Axelson, E. Loaiza, A. D. Bertoldi, G. V. Frana, J. Bryce, J. T. Boerma and C. G. Victora. Equity in maternal, newborn, and child health interventions in Countdown to 2015: A retrospective review of survey data from 54 countries. The Lancet. 2012;379(9822):1225-1233. | 1 |
| 41 | C. A. Corbett and L. C. Callister. Giving birth: The voices of women in Tamil Nadu, India. MCN The American Journal of Maternal/Child Nursing. 2012;37(5):298-305. | 1 |
| 42 | V. Surwade and S. Surwade. Health seeking behavior among residents of underprivileged areas of Gangtok, Sikkim. Indian Journal of Public Health Research and Development. 2012;3(4):230-235. | 1 |
| 43 | P. Chaman. Impact of emergency medical support services on public health delivery system in Goa. BMC Proceedings. 2012;6. | 2 |
| 44 | S. K. Gupta, DK. Pal, R. Tiwari, R. Garg, A. K. Shrivastava, R. Sarawagi, R. Patil, L. Agarwal, P. Gupta and C. Lahariya. Impact of Jannai Suraksha Yojana on institutional delivery rate and maternal morbidity and mortality: An observational study. J Health Popul Nutr. 2012;30(4):464-471 | 1 |
| 45 | K. Jehan, K. Sidney, H. Smith and A. de Costa. Improving access to maternity services: an overview of cash transfer and voucher schemes in South Asia. Reproductive Health Matters. 2012;20(39):142–154 | 2 |
| 46 | K. Sidney, V. Diwan, Z. El-Khatib and A. de Costa. India's JSY cash transfer program for maternal health: who participates and who doesn't--a report from Ujjain district. Reproductive health. 2012;9:2. | 1 |
| 47 | K. D. Jamwal, A. Goel, K. G. Sajith, U. G. Zachariah, J. Ramachandran and C. E. Eapen. Maternal deaths from pregnancy associated liver diseases-under-reported or under-recognized in India. Journal of Clinical and Experimental Hepatology. 2012;1:S33. | 1 |
| 48 | G. R. Babu, S. S. Ramachandra, U. Garikipati, T. Mahapatra, S. Mahapatra, S. Narayana and H. Pant. Maternal health correlates of neonatal deaths in a tribal area in India. Internet Journal of Epidemiology. 2012;10:2. | 1 |
| 49 | P. Chauhan, V. K. Chauhan and P. Shrivastava. Maternal mortality among tribal women at a tertiary level of care in Bastar, Chhattisgarh. Global journal of health science. 2012;4(2):132-141. | 1 |
| 50 | R. Bala, K. P. Devi and C. M. Singh. Maternal near miss: Its trend and role as an indicator of maternal morbidity. International Journal of Gynecology and Obstetrics. 2012;119:S289. | 1 |
| 51 | R. Ved, T. Sundararaman, G. Gupta and G. Rana. Program evaluation of Janani Suraksha Yojana. BMC Proceedings. 2012;6(Suppl 5):O15 | 1 |
| 52 | S. Poornima and M. Vinay. Quality of antenatal care services received by mothers attending Under Fives Clinic at MIMS Hospital, Mandya, Karnataka State, India. Indian Journal of Maternal and Child Health. 2012;14(1):8-10. | 1 |
| 53 | A. Bhowmik, K. Jayanna, L. Avery, Ramesh, P. Mony, A. Thomas, K. Shankar, E. Fischer, S. Moses and J. Blanchard. Quality of postpartum care in the community: Assessment from rural areas of Northern Karnataka, India. International Journal of Gynecology and Obstetrics. 2012;119:S380. | 1 |
| 54 | S. Rani, D. Chawla, A. Huria and S. Jain. Risk factors for perinatal mortality due to asphyxia among emergency obstetric referrals in a tertiary hospital. Indian Pediatrics. 2012;49(3):191-194. | 2 |
| 55 | S. Singh, P. Chhabra and R. Sujoy. Role of Traditional Birth Attendants (TBAs) in Provision of Antenatal and Perinatal Care at Home Amongst the Urban Poor in Delhi, India. Health Care for Women International. 2012;33(7):666-676. | 2 |
| 56 | C. Ameh, S. Msuya, J. Hofman, J. Raven, M. Mathai and N. van den Broek. Status of emergency obstetric care in six developing countries five years before the MDG targets for maternal and newborn health. PLoS ONE. 2012;7:12. e49938. | 1 |
| 57 | U. Bapat, G. Alcock, N. S. More, S. Das, W. Joshi and D. Osrin. Stillbirths and newborn deaths in slum settlements in Mumbai, India: A prospective verbal autopsy study. BMC Pregnancy and Childbirth. 2012;12:39. | 1 |
| 58 | R. Bhattacharyya and A. Pal. Stillbirths in a referral medical college hospital, West Bengal, India: A ten-year review. Journal of Obstetrics and Gynaecology Research. 2012;38(1):266-271. | 2 |
| 59 | S. Chaturvedi and B. Randive. Study of initiatives to address shortage of specialists for emergency obstetric care in Maharashtra, India. BMC Proceedings. 2012;6. | 1 |
| 60 | N. Van Den Broek. The "making it happen" programme-improving the availability and quality of essential obstetric care in LMIC. International Journal of Gynecology and Obstetrics. 2012;119:S254. | 2 |
| 61 | G. Gita, S. Bharti, K. Shashi and K. Arvind. Trends in maternal mortality and impact of Janani Suraksha Yojana (JSY) on Maternal Mortality Ratio in a tertiary referral hospital. The Journal of Obstetrics and Gynecology of India. 2012;62(3):307–311 | 1 |
| 62 | S. Ghike and P. Asegaonkar. Why obstetric patients are admitted to intensive care unit? A retrospective study. Journal of SAFOG. 2012;4(2):90-92. | 1 |
| 63 | N. Partha, A. Lokesh, S. K. Gupta, A. Lokeshmaran and M. Prabakaran. A study on utilization of antenatal care services among rural women in Pondicherry district. Indian Journal of Maternal and Child Health. 2011;13(2):8p. | 1 |
| 64 | S. S. Mumbare and R. Rege. Ante natal care services utilization, delivery practices and factors affecting them in tribal area of North Maharashtra. Indian Journal of Community Medicine. 2011;36(4):287-290. | 1 |
| 65 | N. Rustagi, J. G. Prasuna and M. D. Vibha. Antenatal care and morbidity profile of pregnant women in an urban resettlement colony of Delhi, India. East African journal of public health. 2011;8(2):157-160. | 1 |
| 66 | R. Kumari, V. Mengi, R. Bahl and Kiran. Antenatal care seeking behaviour of pregnant women in a rural area of Jammu. Indian Journal of Maternal and Child Health. 2011;13(3):7p. | 1 |
| 67 | A. L. Montgomery, S. K. Morris, R. Kumar, R. Jotkar, P. Mony, D. G. Bassani and P. Jha. Capturing the context of maternal deaths from verbal autopsies: a reliability study of the maternal data extraction tool (M-DET). PloS one. 2011;6:2. e14637. | 1 |
| 68 | T. S. Beerenahally, U. Bhojani, S. Amruthavalli, R. Devadasan, M. S. Munegowda, C. Anthonyamma and N. Devadasan. Controlled lives: Impact of social factors on reproductive rights of women in urban South India. Tropical Medicine and International Health. 2011;16:299-305. | 1 |
| 69 | I. Hazarika. Factors that determine the use of skilled care during delivery in India: implications for achievement of MDG-5 targets. Maternal and child health journal. 2011;15(8):1381-1388. | 1 |
| 70 | R. V. Deshpande. Is Janani Suraksha Yojana (JSY) contributing to the reduction of maternal and infant mortality? An insight from Karnataka. The Journal of Family Welfare. 2011;57(1):1-9. | 1 |
| 71 | P. Chhabra, G. Sharma and A. T. Kannan. Pattern of maternal health care among working women in Delhi. Indian Journal of Maternal and Child Health. 2011;13(4):9p. | 1 |
| 72 | N. M. Bellows, B. W. Bellows and C. Warren. Systematic Review: the use of vouchers for reproductive health services in developing countries: systematic review. Tropical Medicine & International Health. 2011;16(1):84-96. | 2 |
| 73 | P. Yerpude and K. Jogdand. A study on delivery and newborn care practices in a rural area of South India. Indian Journal of Maternal and Child Health. 2010;12(4):8p. | 1 |
| 74 | S. Khandelwal, M. Dhanaraj and A. Khandelwal. Admission test as precursor of perinatal outcome: a prospective study. Archives of Gynecology & Obstetrics. 2010;282(4):377-82; | 1 |
| 75 | F. A. Zaman and N. B. Laskar. An application of Indian public health standard for evaluation of primary health centers of an EAG and a Non-EAG state. Indian journal of public health. 2010;54(1):36-39. | 1 |
| 76 | S. Agarwal, V. Sethi, K. Srivastava, P. K. Jha and A. H. Baqui. Birth preparedness and complication readiness among slum women in Indore city, India. Journal of health, population, and nutrition. 2010;28(4):383-391. | 2  Top of Form  Bottom of Form |
| 77 | A. Haldar, B. Baur, U. Dasgupta, K. K. Majumdar, S. N. Jha and S. Ghosh. Critical appraisal of healthcare delivery services by multipurpose workers in rural West Bengal. Journal of the Indian Medical Association. 2010;108(11):750-3 | 1 |
| 78 | S. Singh, D. C. Jain and M. Singh. Determinants of utilization of Maternal and Child health services in a rural area of Uttar Pradesh, (India). Indian Journal of Maternal and Child Health. 2010;12(4):6p. | 1 |
| 79 | NIHFW. Evaluation of MAMTA Scheme in National Capital Territory of Delhi. Department of Planning & Evaluation. National Institute of Health and Family Welfare. 2010;New Delhi | 1 |
| 80 | S. Rath, N. Nair, P. K. Tripathy, S. Barnett and S. Rath. Explaining the impact of a women's group led community mobilisation intervention on maternal and newborn health outcomes: the Ekjut trial process evaluation. BMC International Health and Human Rights. 2010;10:25-37. | 1 |
| 81 | A. Pandey and R. Ranjan. Hypertensive disorders in pregnancy requiring emergency (108) transportation in the state of Gujarat (India): An epidemiological study. Journal of Clinical and Diagnostic Research. 2010;4(1):2017-2022. | 2 |
| 82 | A. Aggarwal. Impact evaluation of India's Yeshasvini community-based health insurance programme. Health Economics. 2010;19(Suppl 1):5-35. | 1 |
| 83 | M. E. Khan, A. Hazra and I. Bhatnagar. Impact of Janani Suraksha Yojana on selected family health behaviors in rural Uttar Pradesh. Journal of Family Welfare. 2010;56 Spec No:9-22. | 1 |
| 84 | A. Giovine and C. Ostrowski. Improving Transportation and Referral for Maternal Health: Knowledge Gaps & Recommendations. Technical Report: May 19. 2010; Available at https://www.wilsoncenter.org/sites/default/files/Transport%20Referral%20Meeting%20Technical%20Report.pdf | 2 |
| 85 | Population Council. Increasing institutional delivery and access to emergency obstetric care services in rural Uttar Pradesh: Implications for behavior change communication. 2010;4p. Available at: http://www.popcouncil.org/uploads/pdfs/2010India_PolicyBrief01.pdf | 2 |
| 86 | V. K. Paul. India: conditional cash transfers for in-facility deliveries. The Lancet. 2010;375(9730):1943-1944. | 1 |
| 87 | S S. Lim, L. Dandona, J. A. Hoisington, S. L. James, M. C. Hogan and E. Gakidou. India’s Janani Suraksha Yojana, a conditional cash transfer programme to increase births in health facilities: an impact evaluation. Lancet. 2010;375:2009–23 | 1 |
| 88 | R. Ghosh and A. K. Sharma. Intra- and inter-household differences in antenatal care, delivery practices and postnatal care between last neonatal deaths and last surviving children in a peri-urban area of India. Journal of Biosocial Science. 2010;42(4):511-30. | 1 |
| 89 | M. Chaturvedi, D. Nandan and S. C. Gupta. Mother care practices: A cluster survey. Indian Journal of Public Health Research and Development. 2010;1(1):38-42. | 1 |
| 90 | P. Anita, R. B. Jain, M. S. Punia, R. Vidya and M. Kalhan. Pattern of deliveries in rural areas of a district in Haryana, India. Internet Journal of Epidemiology. 2010;9:1. | 1 |
| 91 | S. Sharma, M. Satija, R. K. Sachar, R. K. Soni and G. P. Singh. Perinatal mortality in multiple pregnancy in a rural area of Punjab. Indian Journal of Maternal and Child Health. 2010;12(1):5p. | 1 |
| 92 | S. Anand and R. K. Sinha. Quality differentials and reproductive health service utilisation determinants in India. International Journal of Health Care Quality Assurance. 2010;23(8):718-729. | 1 |
| 93 | R. S. Rao, G. Lakshmi and R. S. Sravani. Safe motherhood interventions during antenatal period among Koya Dora tribal women. Indian Journal of Maternal and Child Health. 2010;12(3):7p. | 1 |
| 94 | T. Jain, J. V. Singh, M. Bhatnagar, S. Garg and H. Chopra. Status of antenatal care in slums of Meerut City. Indian Journal of Maternal and Child Health. 2010;12(4):7p; | 1 |
| 95 | P. Rajkumar, M. Anuj, K. Ss and P. Kc. Status of maternal health care services after training of grass root health functionaries. Indian Journal of Maternal and Child Health. 2010;12(1):7p. | 2 |
| 96 | M. N. Bhanderi and S. Kannan. Untreated reproductive morbidities among ever married women of slums of Rajkot City, Gujarat: the role of class, distance, provider attitudes, and perceived quality of care. Journal of urban health: bulletin of the New York Academy of Medicine. 2010;87(2):254-263. | 1 |
| 97 | R. K. Jha, S. Gopalakrishnan, K. Ajitha, D. Kuberan and M. M. Rana. Utilization of maternal health care services in Kancheepuram District, Tamil Nadu. Indian Journal of Maternal and Child Health. 2010:12(4):7p. | 1 |
| 98 | S. J. Jejeebhoy, Shveta Kalyanwala, AJ Francis Zavier, Rajesh Kumar and Nita Jha. Experience seeking abortion among unmarried young women in Bihar and Jharkhand, India: delays and disadvantages. Reproductive Health Matters. 2010;18(35):163–174 | 2 |
| 99 | G. Mona, D. Mavlankar, P. Trivedi. A study of referral system for EmOC in Gujarat. . 2009;Available at http://www.iimahd.ernet.in/publications/data/2009-06-02Gupta.pdf | Top of Form  Bottom of Form  2 |
| 100 | D. J. Bhanderi, S. M. Mukherjee, M. K. Gohel and D. S. Christian. An evaluation of the utilisation of reproductive and child health services provided by government to the rural community of Anand District, Gujrat. Indian journal of public health. 2009;53(4):250-252. | 1 |
| 101 | R. K. Srivastava, S. Kansal, V. K. Tiwari, L. Piang, R. Chand and D. Nandan. Assessment of utilization of RCH services and client satisfaction at different level of health facilities in Varanasi District. Indian journal of public health. 2009;53(3):183-189. | 1 |
| 102 | A. Gorthi, C. Firtion and J. Vepa. Automated risk assessment tool for pregnancy care. Conference proceedings: Annual International Conference of the IEEE Engineering in Medicine and Biology Society. IEEE Engineering in Medicine and Biology Society. Conference. 2009;6222-6225. | 2 |
| 103 | A. Pandey and R. Ranjan . Emergency (108) calls to the Ambulance service in the state of Gujarat (India) that do not result in the patient being transported to hospital: An epidemiological study. Journal of Clinical and Diagnostic Research. 2009;3:1519-1522 | 2 |
| 104 | J. Hussein,D. Newlands, L. D’Ambruoso, I. Thaver, R. Talukder and G. Besanaa. Identifying practices and ideas to improve the implementation of maternal mortality reduction programmes: findings from five South Asian countries. British Journal of Obstetrics and Gynaecology. 2009;117:304-313 | 1 |
| 105 | G. Francis Raj. Importance of effective emergency medical transport in addressing maternal complications: Case study, EMRI 108 EMS service in Andhra Pradesh. EMRI Journal. 2009; | 2 |
| 106 | S. Bonu, I. Bhushan, M. Rani and I. Anderson. Incidence and correlates of 'catastrophic' maternal health care expenditure in India. Health Policy and Planning. 2009;24(6):445-456. | 1 |
| 107 | D. V. Mavalankar, K. S. Vora, K. V. Ramani, P. Raman, B. Sharma and M. Upadhyaya. Maternal health in Gujarat, India: a case study. Journal of health, population, and nutrition. 2009;27(2):235-248. | 2 |
| 108 | K. S. Vora, D. V. Mavalankar, K. V. Ramani, M. Upadhyaya, B. Sharma, S. Iyengar, V. Gupta and K. Iyengar. Maternal health situation in India: a case study. Journal of health, population, and nutrition. 2009;27(2):184-201. | 1 |
| 109 | R. Bhat, D. V. Mavalankar, P. V. Singh and N. Singh. Maternal healthcare financing: Gujarat's Chiranjeevi Scheme and its beneficiaries. Journal of health, population, and nutrition. 2009;27(2):249-258;. | 1 |
| 110 | V. Salvi. Maternal mortality in India: The true killers. Journal of Postgraduate Medicine. 2009;55(1):1-2. | 1 |
| 111 | S. K. Mohanty and P. K. Pathak. Rich-poor gap in utilization of reproductive and child health services in India, 1992-2005. Journal of biosocial science. 2009;41(3):381-398. | 1 |
| 112 | D. Mavalankar, A. Singh, SR. Patel, A. Desai and P. V. Singh. Saving mothers and newborns through an innovative partnership with private sector obstetricians: Chiranjeevi scheme of Gujarat, India. International Journal of Gynecology and Obstetrics. 2009;107:271–276 | 1 |
| 113 | N. S. More, G. Alcock, U. Bapat, S. Das, W. Joshi and D. Osrin. Tracing pathways from antenatal to delivery care for women in Mumbai, India: cross-sectional study of maternity in low-income areas. International Health. 2009;1(1):71-77. | 2 |
| 114 | A. Jain, S. C. Gupta, S. K. Misra, R. Singh, A. K. Bhagoliwal and S. K. Kaushal. Trend and causes of maternal mortality among women delivering in S. N. Medical College Hospital, Agra. Indian journal of public health. 2009;53(1):47-48. | 1 |
| 115 | C. L. Evans, D. Maine, L. McCloskey, F. G. Feelay and H. Sanghvi. Where there is no obstetrician- increasing capacity for emergency obstetric care in rural India: an evaluation of a pilot program to train general doctors. International Journal of Gynecology and Obstetrics. 2009;107(3):277-282. | 2 |
| 116 | S. Barnett, N. Nair, P. Tripathy, J. Borghi, S. Rath and A. Costello. A prospective key informant surveillance system to measure maternal mortality - findings from indigenous populations in Jharkhand and Orissa, India. BMC pregnancy and childbirth. 2008;8:6. | 1 |
| 117 | P. Mohan, S. D. Iyengar, K. Agarwal, J. C. Martines and K. Sen. Care-seeking practices in rural Rajasthan: Barriers and facilitating factors. Journal of Perinatology. 2008;28(Supl. 2):S31-S37. | 1 |
| 118 | M. Rani, S. Bonu and S. Harvey. Differentials in the quality of antenatal care in India. International Journal for Quality in Health Care. 2008;20(1):62-71. | 1 |
| 119 | UNICEF. Maternal and Perinatal death enquiry: Empowering communities to avert maternal Deaths in india and response. UNICEF. 2008;New Delhi | 2 |
| 120 | S. K. Bhattacharyya, A. K. Majhi, S. L. Seal, S. Mukhopadhyay, G. Kamilya and J. Mukherji. Maternal mortality in India: A 20-year study from a large referral medical college hospital, West Bengal. Journal of Obstetrics and Gynaecology Research. 2008;34(4):499-503. | 2 |
| 121 | A. H. Baqui, A. M. Rosecrans, E. K. Williams, P. K. Agrawal, S. Ahmed, G. L. Darmstadt, V. Kumar, U. Kiran, D. Panwar, R. C. Ahuja, V. K. Srivastava, R. E. Black and M. Santosham. NGO facilitation of a government community-based maternal and neonatal health programme in rural India: Improvements in equity. Health Policy and Planning. 2008;23(4):234-243. | 1 |
| 122 | P. Chhabra, K. Guleria, N. K. Saini, K. T. Anjur and N. B. Vaid. Pattern of severe maternal morbidity in a tertiary hospital of Delhi, India: A pilot study. Tropical Doctor. 2008:38(4):201-204. | 1 |
| 123 | L. Barrick and M. A. Koenig. Pregnancy intention and antenatal care use in two rural north Indian States. World health & population. 2008;10(4):21-37. | 1 |
| 124 | S. K. Raina, M. Vijay and S. Gurdeep. Trends in antenatal care utilization in block R.S. Pura of district Jammu. JK Practitioner. 2008;15:40-42. | 1 |
| 125 | N. Bhola, R. Kumari and T. Nidha. Utilization of the health care delivery system in a district of North India. East African journal of public health. 2008;5(3):147-153. | 1 |
| 126 | D. Donaldson, H. Sethi and S. Sharma. Vouchers to Improve Access by the Poor to Reproductive Health Services: Design and Early Implementation Experience of a Pilot Voucher Scheme in Agra District, Uttar Pradesh, India. Health Policy Initiative, Task Order 1, Futures Group International.. 2008;Washington, DC | 1 |
| 127 | L. Say and R. Raine. A systematic review of inequalities in the use of maternal health care in developing countries: Examining the scale of the problem and the importance of context. Bulletin of the World Health Organization. 2007;85(10):812-819. | 1 |
| 128 | S. Chaturvedi and B. Ranadive. Are we really making motherhood safe? A study of provision of iron supplements and emergency obstetric care in rural Maharashtra. National Medical Journal of India. 2007;20(6):294-296. | 1 |
| 129 | P. Agarwal, M. M. Singh and S. Garg. Maternal health-care utilization among women in an urban slum in Delhi. Indian Journal of Community Medicine. 2007;32(3):203-205. | 1 |
| 130 | S. Bharati, M. Pal and P. Bharati. Obstetric care practice in Birbhum District, West Bengal, India. International Journal for Quality in Health Care. 2007;19(4):244-249. | 1 |
| 131 | A. Aggarwal, A. Pandey and B. N. Bhattacharya. Risk factors for maternal mortality in Delhi slums: a community-based case-control study. Indian Journal of Medical Sciences. 2007;61(9):517-26. | 1 |
| 132 | R. Jeffery, P. Jeffery and M. Rao. Safe Motherhood Initiatives: Contributions from small-scale studies. Indian Journal of Gender Studies. 2007;14(2):285-294. | 1 |
| 133 | J. Patricia, D. Abhijit, D. Jashodhara and J. Roger. Unmonitored intrapartum Oxytocin use in home deliveries: Evidence from Uttar Pradesh, India. Reproductive Health Matters. 2007;5(30):172–178 | 2 |
| 134 | A. Sinhababu, B. S. Mahapatra, D. Das, M. Mundle, A. B. Soren and T. K. Panja. A study on utilization and quality of coverage of antenatal care services at the subcentre level. Indian journal of public health. 2006;50(1):49-52. | 1 |
| 135 | N. T. Ngoc, M. Merialdi, H. Abdel-Aleem, G. Carroli, M. Purwar, N. Zavaleta, L. Campodonico, M. M. Ali, G. J. Hofmeyr, M. Mathai, O. Lincetto and J. Villar. Causes of stillbirths and early neonatal deaths: data from 7993 pregnancies in six developing countries. Bulletin of the World Health Organization. 2006;84(9):699-705. | 1 |
| 136 | N Chandhiok, B. S. Dhillon, I. Kambo and N. C. Saxena. Determinants of antenatal care utilization in rural areas of India : A cross-sectional study from 28 districts (An ICMR task force study). The Journal of Obstetrics and Gynecology of India. 2006;56(1):47-52 | 1 |
| 137 | R. K. Sinha, S. K. Mohanty, T. K. Roy and M. Koenig. Do home visits by health workers make a difference in service utilization? Findings from a longitudinal study in rural India. Demography India. 2006;35(2): 219-232. | 1 |
| 138 | T. S. Sunil, S. Rajaram and L. K. Zottarelli. Do individual and program factors matter in the utilization of maternal care services in rural India? A theoretical approach . . 2006;Available at http://demoscope.ru/weekly/knigi/tours_2005/papers/iussp2005s50607.pdf | 2 |
| 139 | F. Ram and A. Singh. Is antenatal care effective in improving maternal health in rural Uttar Pradesh? Evidence from a district level household survey. Journal of biosocial science. 2006;38(4):433-448. | 1 |
| 140 | A. H. Khosla, K. Dahiya and K. Sangwan. Maternal mortality in eclampsia: 489 cases. Tropical Doctor. 2006;36(1):47-9. | 2 |
| 141 | N. Gupta, S. Kumar, N. C. Saxena, D. Nandan and B. N. Saxena. Maternal mortality in seven districts of Uttar Pradesh--an ICMR task force study. Indian journal of public health. 2006;50(3):173-178. | 2 |
| 142 | C. Ronsmans and W. J. Graham. Maternal mortality: who, when, where, and why. Lancet. 2006;368(9542):1189-1200. | 1 |
| 143 | P. Chhabra, A. K. Sharma and K. A. Tupil. Obstetric and neonatal outcomes in women who live in an urban resettlement area of Delhi, India: A cohort study. Journal of Obstetrics and Gynaecology Research. 2006;32(6):567-573. | 1 |
| 144 | S. Sinha. Outcome of antenatal care in an urban slum of Delhi. Indian Journal of Community Medicine. 2006;31(3):189-191. | 1 |
| 145 | M. K. Choe and J. Chen. Potential for reducing child and maternal mortality through reproductive and child health intervention programmes: an illustrative case study from India. Asia-Pacific Population Journal. 2006;21(1):13-44. | 1 |
| 146 | A. H. Baqui, G. L. Darmstadt, E. K. Williams, V. Kumar, T. U. Kiran, D. Panwar, V. K. Srivastava, R. Ahuja, R. E. Black and M. Santosham. Rates, timing and causes of neonatal deaths in rural India: implications for neonatal health programmes. Bulletin of the World Health Organization. 2006;84(9):706-13. | 1 |
| 147 | S. W. Mercer, K. Sevar and T. D. Sadutshan. Using clinical audit to improve the quality of obstetric care at the Tibetan Delek Hospital in North India: A longitudinal study. Reproductive Health. 2006;3:4. | 2 |
| 148 | K. Guleria, S. Bansal, N. Agarwal and V. Grover. Women with Septic Abortion: Who, How and Why? A prospective study from tertiary care hospital in India. Indian Journal of Public Health. 2006;2:95-96 | 2 |
| 149 | A. B. Biswas, D. K. Das, R. Misra, R. N. Roy, D. Ghosh and K. Mitra. Availability and use of emergency obstetric care services in four districts of West Bengal, India. Journal of Health, Population and Nutrition. 2005;23(3):266-274. | 2 |
| 150 | S. Haider, V. Kashyap and P. E. Soren. Awareness regarding antenatal services among ANMs in Shikaripara PHC of Dumka Dist. Jharkhand. Indian Journal of Preventive and Social Medicine. 2005;36(4):78-82. | 2 |
| 151 | S. Kumar. Challenges of maternal mortality reduction and opportunities under National Rural Health Mission - a critical appraisal. Indian Journal of Public Health. 2005:49(3):163-167. | 2 |
| 152 | J. de Graft-Johnson, P. Daly, S. Otchere, N. Russell and R. Bell. Household-to-hospital continuum of maternal and newborn care. 2005;12p. Available at http://www.coregroup.org/storage/documents/Workingpapers/HHCC_screen.pdf | 2 |
| 153 | Judith T. Fullerton, Richard Killian and Patricia M. Gass. Outcomes of a Community– and Home-Based Intervention for Safe Motherhood and Newborn Care. Health Care for Women International. 2005;26;7:561-576. DOI:10.1080/07399330591004881 | 2 |
| 154 | A. Ager and K. Pepper. Patterns of health service utilization and perceptions of needs and services in rural Orissa. Health policy and planning. 2005;20(3):176-184. | 1 |
| 155 | S. Chhabra, A. Kaipa and A. Kakani. Reduction in maternal mortality due to sepsis. Journal of Obstetrics and Gynaecology. 2005;25(2):140-142. | 1 |
| 156 | D. Vidyasagar. Regionalization of perinatal care: Its relevance to India and other developing countries. Journal of Neonatology. 2005;19(4):293-303. | 1 |
| 157 | B. Joseph, S. Charles, T. J. Prakash, M. L. Sudan and G. Jasmine. Utilization of antenatal services in apparel manufacturing factories in Bangalore. Indian Journal of Occupational and Environmental Medicine. 2005;9(3):107-110. | 1 |
| 158 | S. Pallikadavath, M. Foss and R. W. Stones. Antenatal care: Provision and inequality in rural north India. Social Science and Medicine. 2004;59(6):1147-1158. | 1 |
| 159 | S. Chhabra and R. Sirohi. Averting maternal deaths in spite of resource constraints: An Indian rural experience over two decades. Journal of Obstetrics and Gynaecology. 2004;24(5):521-524. | 2 |
| 160 | R. Stephenson and Z. Matthews. Maternal health-care service use among rural-urban migrants in Mumbai, India. Asia-Pacific Population Journal. 2004;19(1):39:60. | 1 |
| 161 | R. A. Bang, A. T. Bang, M. H. Reddy, M. D. Deshmukh, S. B. Baitule and V. Filippi. Maternal morbidity during labour and the puerperium in rural homes and the need for medical attention: A prospective observational study in Gadchiroli, India. BJOG: An International Journal of Obstetrics and Gynaecology. 2004;111(3):231-238. | 2 |
| 162 | M. K. Baul and Manjusha. Maternal mortality--a ten-year study. Journal of the Indian Medical Association. 2004;102(1):18-19. | 2 |
| 163 | Population Council. The SEARCH Experience: quality reproductive health services in rural India -- India. Health for the Millions. 2004:87-88; Available at http://www.popcouncil.org/uploads/pdfs/2010India_PolicyBrief01.pdf | 2 |
| 164 | S. Chhabra and R. Sirohi. Trends in maternal mortality due to haemorrhage: Two decades of Indian rural observations. Journal of Obstetrics and Gynaecology. 2004;24(1):40-43. | 2 |
| 165 | A. M. Vaijyanath and S. Mittal. Antenatal care assessment in a rural and a tertiary hospital by obstetric risk scoring system. Journal International Medical Sciences Academy. 2003;16(4):189-190. | 1 |
| 166 | R. Cheema, T. S. Cheema and K. Kaushal. Correlation between ANC and safe motherhood in northern India. Journal of Obstetrics, Gynaecology and Family Welfare. 2003:5-18; | 1 |
| 167 | S. C. Mohapatra and P. Mohapatra. Rendering effective RCH care: handling emergencies in obstetrical complications. Indian Journal of Preventive and Social Medicine. 2003;34(3-4):153-159. | 2 |
| 168 | ME. Khan. Situation analysis of the postpartum programme in India. National Medical Journal of India. 2003;16(Suppl.2):28-34. | 2 |
| 169 | S. Agarwal and I. Sarasua. Community-based health financing: CARE India's experience in the maternal and infant survival project. Research in Healthcare Financial Management. 2002;7(1):85-94. | 1 |
| 170 | R. Stephenson and A. O. Tsui. Contextual influences on reproductive health service use in Uttar Pradesh, India. Studies in Family Planning. 2002;33(4):309-320. | 1 |
| 171 | AMDD working group. Program note: Using UN process indicators to assess needs in emergency obstetric services: Bhutan, Cameroon and Rajasthan, India. International Journal of Gynecology and Obstetrics. 2002;77(3):277-284. | 1 |
| 172 | S. Chhabra and M. Dhorey. Retained placenta continues to be fatal but frequency can be reduced. Journal of Obstetrics & Gynaecology. 2002;22(6):630-3. | 1 |
| 173 | K. Navaneetham and A. Dharmalingam. Utilization of maternal health care services in Southern India. Social Science and Medicine. 2002;55(10):1849-1869. | 1 |
| 174 | P. Pandey, S. Mishra, M. Alwani and R. Shukla. Anaemia a major killer of pregnant women in Jabalpur. Tribal Health Bulletin. 2001;7(2):1-5. | 1 |
| 175 | R. N. Sinha, S. Dasgupta, D. Pal, N. K. Mondal and P. R. Karmakar. Coverage of maternal care services in the state of West Bengal. Indian Journal of Public Health. 2001;45(4):116-21. | 1 |
| 176 | S. S. Bloom, D. Wypij and M. Das Gupta. Dimensions of women's autonomy and the influence on maternal health care utilization in a north Indian city. Demography. 2001;38(1):67-78. | 1 |
| 177 | Arvind Sehgal, M.S. Roy, N.K. Dubey and M.C. Jyothi. Factors contributing to outcome in newborns delivered out of hospital and referred to a teaching institution. Indian pediatrics. 2001;38(11):1289-1294. | 1 |
| 178 | J. C. Bhatia and J. Cleland. Health-care seeking and expenditure by young Indian mothers in the public and private sectors. Health Policy and Planning. 2001;16(1):55-61. | 1 |
| 179 | K. S. Sugathan, V. Mishra and R. D. Retherford. Promoting institutional deliveries in rural India: the role of antenatal-care services. National Family Health Survey Subject Reports Number 20. IIPS. Mumbai. 2001:38p. Available at http://www.eastwestcenter.org/sites/default/files/filemanager/Research_Program/NFHS_Subject_Reports/subj-20.pdf | 1 |
| 180 | P. Singh and R. J. Yadav. Status of ante-natal coverage in four states. Health and Population: Perspectives and Issues. 2001;24(3):148-156. | 1 |
| 181 | T. K. Sundari Ravindran and U. S. Mishra. Unmet need for reproductive health in India. Reproductive Health Matters. 2001;9(18):105-13. | 1 |
| 182 | U. Chatterjee and K. B. Saha. An overview of utilization of antenatal care services among the scheduled tribe of major states in India. Anthropologist. 2000;2(1):37-42. | 1 |
| 183 | P. Singh and R. J. Yadav. Antenatal care of pregnant women in India. Indian Journal of Community Medicine. 2000;7p. | 1 |
| 184 | D. Shah, S. Shroff and K. Ganla. Factors affecting perinatal mortality in India (perinatal audit). Prenatal and Neonatal Medicine. 2000;5(5):288-302. | 2 |
| 185 | K. Navaneetham, and A. Dharmalingam. Utilisation of maternal health care services in South India. 2000;Available at http://unpan1.un.org/intradoc/groups/public/documents/APCITY/UNPAN012686.pdf | 1 |
| 186 | S. S. Bloom, T. Lippeveld and D. Wypij. Does antenatal care make a difference to safe delivery? A study in urban Uttar Pradesh, India. Health Policy & Planning. 1999;14(1):38-48. | 1 |
| 187 | F. Fazili and G. M. Mattoo. Epidemiology of perinatal mortality a hospital based study. JK Practitioner. 1999;6(1):41-45. | 1 |
| 188 | E. Fatula. Lakshmiben: a case study of a "near miss" obstetric event, Gujarat, India. In: Safe Motherhood initiatives: critical issues, edited by Marge Berer and TK Sundari Ravindran. Oxford, England, Blackwell Science. 1999;155-6. | 2 |
| 189 | V. Pendse. Maternal deaths in an Indian hospital: a decade of (no) change? In: Safe Motherhood initiatives: critical issues, edited by Marge Berer and TK Sundari Ravindran. Oxford, England, Blackwell Science. 1999;119-26. | 1 |
| 190 | R. A. Ansari and B. C. Patel. Quality of reproductive health services at community health centres. Research summary. 1999:2p. Available at http://www.cortindia.in/RS%5CRS-1998-0302.pdf. | 2 |
| 191 | B. B. Nielsen, M. Hedgaard, S. H. Thilsted, A. Joseph and J. Liljestrand. Does antenatal care influence postpartum health behaviour? Evidence from a community based cross-sectional study in rural Tamil Nadu, south India. British journal of obstetrics and gynaecology. 1998;105(7):697-703. | 1 |
| 192 | M. S. Kramer, J. Haas and A. Kelly. Maternal anthropometry-based screening and pregnancy outcome: a decision analysis. Tropical Medicine & International Health. 1998;3(6):447-53. | 1 |
| 193 | L. Behl, N. Grover and S. L. Kaushik. Perinatal and neonatal mortality--a hospital based study. Indian Pediatrics. 1998;35(7):683-4. | 1 |
| 194 | N. Gupta, K. K. Jani, S. Kumari and M. Sood. Early neonatal morbidity and mortality in 'at-risk' and 'normal' term pregnancies. Indian Journal of Pediatrics. 1997;64(4):523-7. | 2 |
| 195 | M. Singh and V. K. Paul. Maternal and child health services in India with special focus on perinatal services. Journal of perinatology: official journal of the California Perinatal Association. 1997;17(1):65-69. | 1 |
| 196 | N. Kavitha and N. Audinarayana. Utilisation and determinants of selected MCH care services in rural areas of Tamil Nadu. Health and Population: Perspectives and Issues. 1997;20(3):112-25. | 1 |
| 197 | A. G. Khan, N. Roy and S. Sureender. Utilisation of reproductive health services in rural Maharashtra. Journal of family welfare. 1997;43(1):37-44. | 1 |
| 198 | U. K. Singh, S. P. Srivastava, A. Kumar, A. K. Thakur, R. Prasad and B. Chakrabarti. Comparative study of perinatal mortality and morbidity in the community and at Medical College Hospital, Patna. Indian pediatrics. 1996;33(12):1057-1058. | 1 |
| 199 | M. Ravikumara and B. V. Bhat. Early neonatal mortality in an intramural birth cohort at a tertiary care hospital. Indian journal of pediatrics. 1996;63(6):785-789. | 1 |
| 200 | S. Thomas and J. Ponnaiya. Fetal and neonatal deaths in a South Indian hospital. Journal of Tropical Pediatrics. 1996;42(2):117-18. | 1 |
| 201 | R. K. Gupta and A. Kumar. Maternal care among slum dwellers in Delhi. Indian journal of maternal and child health. 1996;7(3):78-81. | 1 |
| 202 | J. C. Bhatia and J. Cleland. Obstetric morbidity in South India: Results from a community survey. Social Science and Medicine. 1996;43(10):1507-1516. | 1 |
| 203 | P. Rajaram, A. Agrawal and S. Swain. Determinants of maternal mortality: a hospital based study from south India. Indian journal of maternal and child health. 1995;6(1):7-10. | 1 |
| 204 | S. Nirupam and E. A. Yuster. Emergency obstetric care: Measuring availability and monitoring progress. International Journal of Gynecology and Obstetrics. 1995;50(Suppl. 2):S79-S88. | 2 |
| 205 | S. Mudller. Midwifery in rural India: a study of traditional birth attendants in Tamil Nadu, India. Australian College of Midwives Incorporated journal. 1995;8(1):24-30. | 1 |
| 206 | M. Pradeep, L. Rajam and P. Sudevan. Perinatal mortality--a hospital based study. Indian Pediatrics. 1995;32(10):1091-4. | 1 |
| 207 | V. Srinivasan, S. Radhakrishna, R. Sudha, M. V. Malathi, S. Jabbar, R. Ramakrishnan and T. Venkata Rao. Randomised controlled field trial of two antenatal care packages in rural south India. Indian Journal of Medical Research. 1995;102(8):86-94. | 1 |
| 208 | S. K. Trivedi and A. Khanna. Study of causes of maternal mortality at sub-district level. Health and Population: Perspectives and Issues. 1995;18(1):37-44. | 1 |
| 209 | B.K. Chakravarty. An up-to-date assessment of maternity care programme in the largest miners' colony in Asia. Journal of the Indian Medical Association. 1994;92(5):147-154. | 1 |
| 210 | S. Barge, I. Khan, M. Kini, S. Patel and S. Kumber. Barriers to emergency obstetric care in rural Gujarat. Research summary. 1994;2p. Available at www.cortindia.in/RS%5CRS-1993-06.pdf | 2 |
| 211 | B. Chalmers and D. Meyer. Companionship in the perinatal period. A cross-cultural survey of women's experiences. Journal of Nurse-Midwifery. 1994;39(4):265-72. | 1 |
| 212 | R. Arora, R. P. Ganguli, S. Swain, A. Oumachigui and P. Rajaram. Determinants of maternal mortality in eclampsia in India. Australian and New Zealand Journal of Obstetrics and Gynaecology. 1994;34(5):537-539. | 2 |
| 213 | N. K. Tyagi, M. S. Bharambe, B. S. Garg, J. S. Mathur and K. Goswami. Epidemiology of early neonatal mortality. Indian journal of maternal and child health. 1994;5(4):99-102. | 1 |
| 214 | P. S. Rao and A. Amalraj. Maternal mortality in southern India. Tropical & Geographical Medicine. 1994;46(5):302-4. | 1 |
| 215 | S. K. Kapoor, K. Anand and G. Kumar. Risk factors for stillbirths in a secondary level hospital at Ballabgarh, Haryana: a case control study. Indian Journal of Pediatrics. 1994;61(2):161-6. | 1 |
